# Hydrophobic Interactions in Aqueous Osmolyte Solutions: Thermodynamics of Solvation and Implication on Protein Stability

**DOI:** 10.1021/acs.jpcb.5c00785

**Published:** 2025-05-19

**Authors:** Cedrix J. Dongmo Foumthuim

**Affiliations:** Department of Chemical Sciences (DisC), University of Padova, via Marzolo 1, Padova 35131 Italy

## Abstract

The effect of cosolvents urea and trimethylamine-*N*-oxide (TMAO) on hydrophobic association mechanisms is investigated by employing molecular dynamics simulations and free energy calculations. Three nonpolar moieties are used to model the hydrophobic interactions: *n*-hexane *n*C_6_H_14_, neopentane C_5_H_12_, and cyclohexane cC_6_H_12_. These hydrophobic model systems are subsequently immersed in four different solvent models with varied composition: pure water, aqueous urea, aqueous TMAO, and mixed urea-TMAO ternary solution. The solute–solute potentials of mean force (PMF), solute-water, and solute-cosolvent distribution functions are reported. Both urea and TMAO are found to have only moderate effects on hydrophobic associations, thereby mainly acting as glue bridging between pairwise hydrophobic moieties holding them together. Furthermore, it is seen that TMAO mediates the formation of hydrogen bonds between its oxygen atom and water or urea while still favoring the hydrophobic contacts with the hydrophobic surface, thereby acting as a kind of amphiphile displacing water or urea from the inner solvation shell of the hydrophobic solutes investigated here to the bulk. The analyses of the enthalpic and entropic contributions to PMFs indicate that configurations at the contact minimum are both enthalpically and entropically favorable, though, with a large entropic contribution, whereas solute-separated minimum configurations are dominantly enthalpically driven, induced by stabilizing water hydrogen bonding. To provide a more factual and general perspective to the simplistic hydrophobic models, simulations are also performed on a realistic-like hydrophobic model, β2-microglobulin (β2m), a paradigmatic protein model for amyloid studies. Results show that TMAO protects the β2m folded state by its strong preferential exclusion from the close vicinity of its surface. Contrariwise, urea moieties likely accumulate at the protein surface, thereby displacing water molecules from the hydration shell to the bulk, thus promoting an unfolded state of the protein.

## Graphical abstract

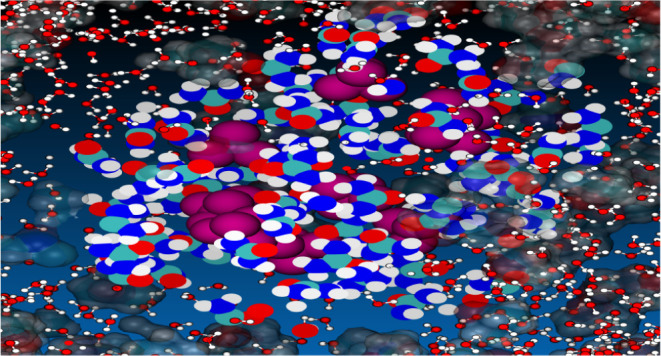

## 1 Introduction

Proteins are functional soft matter moieties of biological systems made up of monomeric blocks of amino acid residues. *In vivo* in general and in solution in particular, they may often adopt different and unique folds, owing to their conformational freedom. However, in many cases, their functionality is optimized in the near-native state, dependent upon the constituted amino acid primary sequence and within a defined range of thermodynamic states, for instance, pH, pressure, temperature, solvent chemical potential, etc.^1^ Indeed, inasmuch as solvent is concerned, its composition and concentration, also including cosolvents present in the cellular compartment, may steer protein conformational stability and proper functioning. Noteworthy, in aqueous solutions, inner solvation shell ∼ 5Å from the protein surface builds a sharp cage of hydrogen-bonded networks, forming the hydration layer, thereby ensuring the hydrodynamics balance with the bulk phase to maintain the structural integrity of proteins.^2^

The (un)­folding thermodynamics balance of proteins can be switched by the presence of cosolvents known as low molecular weight organic compounds affecting the dynamics, stability, and solubility of proteins,^3^ thereby maintaining cellular homeostasis.^4^ Cosolvents driving the equilibrium toward the folded state of proteins are termed protecting osmolytes; meanwhile, those promoting unfolded conformational ensembles are referred to as denaturants. Among the plethora of known colsolvents, urea and trimethylamine *N*-oxide (TMAO) are commonly studied due to their mutually neutralizing effects.^5,6^ Indeed, these latter potentially instigate protein conformational transition in opposite pathways, making them an ideal pair of osmolytes for investigating small molecules pairwise hydrophobic association^7–9^ and/or osmolyte-induced protein (un)­folding.^10–13^ In the present work, our aim is primarily to provide a detailed analysis of the nature of interactions and the influence of urea and TMAO on a small cluster of hydrophobic solutes taken as hydrophobic models.

TMAO is a naturally occurring osmolyte present in the tissues of deep-sea animals wherein it plays many roles including maintaining the hydrostatic pressure.^4^ It is a protective osmolyte known to preserve the folded state of proteins. Although several mechanisms have been proposed to explain this conservative effect, common schemes agree for the preferential exclusion of TMAO from the protein surface or preferential hydration of the protein.^14^ Indeed, TMAO is established to promote the folding of proteins like acetylcholinesterase, myoglobin, α-synuclein, lactate dehydrogenase, etc (see Giri et al.^4^ and references therein). Furthermore, intriguingly, TMAO can also act as a destabilizer for a range of proteins including lysozyme at specific conditions.^15^ Contrariwise, urea is a nonprotecting osmolyte whose action at elevated concentration denatures the folded state of proteins. Nonetheless, albeit acting as a chemical denaturant, urea is found in high concentrations in many species, such as amphibians, marine elasmobranchs, and mammalian kidneys.^16^ Recurrent viable and complementary urea-steered denaturation mechanisms comprise an indirect pathway along which urea reduces hydrophobic interactions through alterations of water structure and a direct interacting scheme via hydrogen bonding with the peptide backbone, see Sarma and Paul^7^ and references therein. This latter mechanism is akin to the preferential binding of urea moieties at the hydrophobic protein surface.

Hydrophobic interactions are thought to be one of the dominant stabilizing forces in biomolecular processes in general and in the folding of proteins in particular.^17–20^ Commonly, these interactions are steered by intramolecular interactions and by the requirement of maximizing the solvent entropy whose combination overwhelms the solute–solvent contacts which may promote expansion or unfolding.^21^ Similarly, the hydrophobic interactions corroborate well with the poor solvent character for a synthetic polymer, mimicking its propensity to aggregate into a compact conformation because the effective intrachain interactions occurring between different monomers composing the polymer overcome the monomer–solvent interactions.^22^ Undoubtedly, these interactions are mediated via an aqueous solvent phase, implying that the stability of the solute is dependent upon the solvent composition and the external thermodynamic states considered, such as the ionic strength, the pressure, the temperature, the solvent chemical potential, the presence of cosolvents, and so forth. In the first part of this work, as mentioned above, the effects of TMAO and urea on the hydrophobic interactions are pertained on three selected hydrophobic models comprising neopentane C_5_H_12_, cyclohexane cC_6_H_12_, and *n*-hexane *n*C_6_H_14_. However, to gather a more general picture and complex hydrophobic interaction mechanisms, a realistic hydrophobic model, β2-microglobulin, is further studied under nearly similar conditions.

β2m is a 99-residue subunit of the major histocompatibility complex class I (MHC I). With a molecular mass of about 12 kDa, it is a small β-sandwich globular protein interacting noncovalently with the human leukocyte antigen HLA-A2 through its α-chain. Thus, the conformation of the α-chain is dependent on the presence of β2m. Therefore, being the subunit of MHC I, its biological role appears to be more structural. Upon dissociation from MHC I, β2m is released in the blood and is essentially cleared by glomerular filtration followed by proximal tubular (in the kidneys) reabsorption and catabolism.^23,24^ In renal insufficient patients undergoing long-term dialysis, it is responsible for dialysis-related amyloidosis (DRA) where insoluble amyloid fibrils of the protein are deposited in joints and connective tissue.^25–27^ The secondary structure of β2m consists of seven β-strands A to G (see Figure SXI^28^) assembled into two antiparallel pleated β-sheets (of 3 + 4 β-strands) connected by a central disulfide bridge (linking strands B and F) highly resembling a β-sandwich immunoglobulin-like type (Ig) C1 domain.^29–31^ No transmembrane domain is found in its structure, and it holds a characteristic molecular assembly called a constant-1 Ig superfamily domain shared with other adaptative immune molecules including MHC I and II.^32^

β2m is mainly responsible for dialysis-related amyloidosis (DRA). DRA is a common incidence of both chronic hemodialysis and peritoneal dialysis, resulting from the increase in the protein level in the serum of patients affected by renal dysfunction. This abnormal increase in protein concentration leads to the maturation of amyloid fibrils that accumulate principally in the osteoarticular tissues (ligaments, bone, muscle, etc.) and viscera, causing organ dysfunctions like carpal tunnel syndrome and bone cysts. It is worth noting that at pH 7, the β2m structure is well folded and does not spontaneously form amyloids,^33,34^ albeit its concentration is steady high in patients undergoing long-term hemodialysis.^25^ Therefore, the extrinsic factors that potentially trigger β2m amyloid formation must be investigated.

Dilip.H.N. and Chakraborty^35^ performed classical molecular dynamics simulations to gain detailed insights on the protein stability induced by aqueous multiphase solutions of TMAO, urea, and their combination using alanine, glycine, *N*-methyl acetamide (NMA), and acetamide as model systems. Their results shed new light on the molecular pathway by which TMAO enhances protein stability but acts oppositely in the presence of urea. This was supported by the strong TMAO-water hydrogen bonding, which indeed strengthens the hydrogen bond lifetime and hydration shell of the system. The model systems chosen in their study are well representative of protein building blocks, so that conclusions drawn therein could straightforwardly mimic the case of realistic protein models. Nonetheless, we point out that their work laid more emphasis on the cosolvent interaction patterns rather than solute–solute association mechanisms, which is instead one of the main goals of our work. Similarly, Su and Dias^36^ used the same cosolvents, though with a different simulation approach but on model peptides to provide rationale of the induced effects of these cosolvents on protein structures. Their results suggest that urea weakens mainly hydrophobic and intrabackbone interactions, while TMAO disentangles hydrophobic interactions and preserves charge–charge and intrabackbone interactions. Contrariwise, in the present study, the hydrophobic models chosen to study the hydrophobic association thermodynamics and pathways are simplistic hydrocarbon moieties. The optimum choice would have been considering amino acid building blocks and/or peptide models, more closely matching realistic protein features. However, the choice made in the current work kind of combine synthetic-like and biopolymers traits as a follow up of our previous works on solvation properties of biomolecules in different environments^20,22^ on one side and synthetic polymers on the other side,^21^ thereby providing a more general and complementary perspective.

Sarma and Paul^7^ used molecular dynamics simulations to investigate the influence of urea and TMAO on the hydrophobic interactions of neopentane C_5_H_12_ moieties build with two distinct models. They find common and disparate features among the different models employed. Namely, they noticed a dehydration pattern of neopentane C_5_H_12_ along with its preferential solvation by urea and TMAO over water for both models used. Furthermore, an anisotropic orientation of water molecules was evidenced near the hydrophobic surface. The present study builds on this work and extends it in several aspects:

- First, two additional analogous six hydrophobic carbon centers, cyclohexane cC_6_H_12_ and *n*-hexane nC_6_H_14_, are investigated here, to cope with our recent curiosity on shape-induced stability character of these latter;^21^
- Second, the temperature dependence of the potential of mean force (PMFs) and the corresponding enthalpic and entropic contributions is provided;
- Third, the solvation free energy in various solvent compositions and its temperature dependence is investigated;
- Fourth, a more general and complete outlook to the strength of hydrophobic interactions and conformation-induced effects of cosolvents is provided by considering a more realistic hydrophobic model, β2-microglobulin protein, a paradigmatic protein model for the study of amyloids.

The article is then organized as follows: After presenting all the necessary technical machinery in Section 2, we present our findings in Section 3 with several subsections dedicated to all the different aspects explored, and finally, we present some take home messages in Section 4.

## 2 Materials and Methods

### 2.1 Potentials of Mean Force (PMF)

We investigated the effects of urea and TMAO on the hydrophobic collapse when a bunch of nonpolar solutes are solvated in an aqueous solution. Three different but representative hydrophobic models have been considered: one linear extended aliphatic alkane *n*-hexane nC_6_H_14_ ; one substituted aliphatic and commonly studied alkane neopentane C_5_H_12_; and one cyclic hydrocarbon cyclohexane cC_6_H_12_, see Figure 1. These hydrophobic model systems were subsequently immersed in four different solvent models with varied composition: pure water, aqueous urea (water-8.16 M urea), aqueous TMAO (water-3.48 M TMAO), and mixed urea-TMAO ternary solution (water-7.18 M urea- 2.87 M TMAO) as described in Table 1. In all the simulations carried out herein, the TIP3P water model^37^ was employed, while the parameters for urea and TMAO were retrieved from the work of Weerasinghe and Smith (KBFF model)^38^ and Kast et al. (Kast model),^39^ respectively. It should be highlighted that our simulations were all performed using flexible models of urea and TMAO, unlike many reported works done by employing a rigid body.^7,8,40^ Moreover, the hydrocarbon hydrophobic models were all modeled in their united atom representation given rise either to a five- or six-site molecular model for neopentane C_5_H_12_ and cyclohexane cC_6_H_12_/*n*-hexane *n*C_6_H_14_, respectively. While the parameters for cyclohexane and *n*-hexane conform to our previous setup,^20–22^ those of neopentane were retrieved from the works of Martin and Siepmann^41^ and Jorgensen et al.^42^ All the atomistic MD simulations were performed with the Gromacs (version 2018.4 and 2022.3) molecular package.^43^

**Figure 1:**
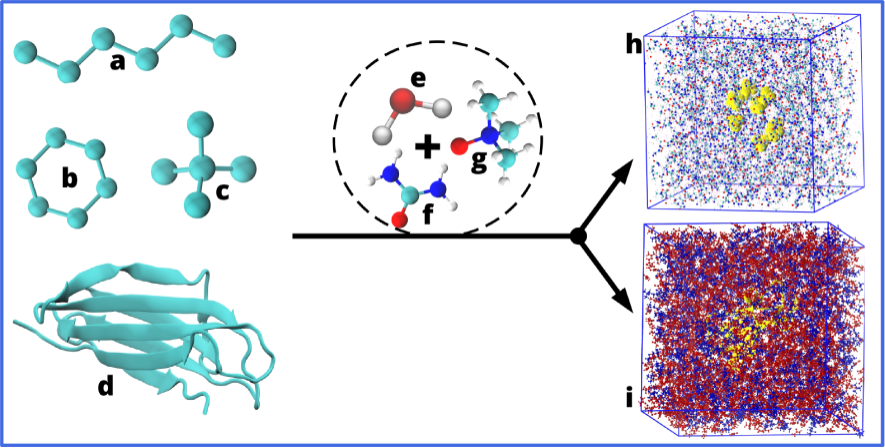
Overview of the studied systems. The hydrophobic solute models considered herein are shown in the left-hand side and correspond respectively to *n*-hexane nC_6_H_14_ (a), cyclohexane cC_6_H_12_ (b), neopentane C_5_H_12_ (c), and β2m (d). Each of them is solvated in different solvent mixtures of water H_2_O (e), urea CO­(NH_2_)_2_ (f), and/or TMAO (CH_3_)_3_NO (g). The resulting systems consisting of 10 hydrodrophic solutes (h) or β2m (i) immersed in the ternary water-7.18 M urea-2.87 M TMAO solution are shown as an illustration. In (h) and (i), water is omitted for clarity, and the solutes are shown in yellow.

**Table 1:** Summary of the Simulated Systems; *n* is the Number of Molecules of Each Simulated Entities with Subscripts s, u, t, and w Representing the Solute (Either Neopentane or Cyclohexane or *n*-Hexane or β2m), Urea, TMAO, and Water, Respectively^a^ ^b^

Systems	Cosolvents	*l* (Å)	*n*_s_	*n*_u_	*n*_t_	*n*_w_	*C*_u_	*C*_t_	*t* (*ns*)
neopentane	water	49.6	10	0	0	3990	0	0	350
	water-urea	54.6	10	800	0	3190	8.16	0	350
	water-TMAO	53.6	10	0	323	3667	0	3.48	350
	water-urea-TMAO	57.0	10	801	320	2869	7.18	2.87	350
cyclohexane	water	49.6	10	0	0	3990	0	0	350
	water-urea	54.6	10	800	0	3190	8.16	0	350
	water-TMAO	53.6	10	0	323	3667	0	3.48	350
	water-urea-TMAO	57.0	10	801	320	2869	7.18	2.87	350
*n*-hexane	water	49.6	10	0	0	3990	0	0	350
	water-urea	54.6	10	800	0	3190	8.16	0	350
	water-TMAO	53.6	10	0	323	3667	0	3.48	350
	water-urea-TMAO	57.0	10	801	320	2869	7.18	2.87	350
β2m	water	80.0	1	0	0	16340	0	0	200
	water-urea	80.0	1	2517	0	8547	8.16	0	200
	water-TMAO	80.0	1	0	1073	11038	0	3.48	200
	water-urea-TMAO	80.0	1	2215	885	5855	7.18	2.87	200

a C represents the molarity of cosolvents (in mol·L^–1^) with subscripts u and t referring to urea and TMAO, respectively.

b l is the cubic simulation box unit cell (in Å). t is the elapsed simulation time (in ns).

In view of deciphering the interaction modes when two nonpolar moieties approach each other, thereby characterizing the extent of their hydrophobicity, we computed the potential of mean force (PMF), *W­(r),* of each of these moieties. The setup employed follows the methodology described by Sarma and Paul^7,8^ in which a system comprising 10 molecules of each hydrocarbon, parametrized in a united-atom like model representation, was randomly inserted into a cubic box. Subsequently, TIP3P water solvents were added to fill the simulation box. Furthermore, to get molecular insightful knowledge on the effects of osmolytes on the hydrophobic interactions, urea and TMAO cosolvents and their combination were added to pure water, leading to four different simulated systems: pure water, binary water-urea and water-TMAO, and ternary mixture water-urea-TMAO. Three models of hydrophobic molecules were considered: neopentane C_5_H_12_, cyclohexane cC_6_H_12_, and *n*-hexane *n*C_6_H_14_. In all the cases, however, the total number of different molecule’s types was 4000. The system’s composition and the Lennard-Jones nonbonded parameters are shown in Tables 1 and 2 below.

**Table 2:** Lennard-Jones Nonbonded Simulation Parameters and Charges Used to Model the Cosolvents Urea and TMAO and Water Moieties

Systems	Atom type	σ (nm)	ϵ (kJmol^–1^)	charge (*e*)
neopentane	C(central)	0.3800	0.2092	0
	C (CH_3_)	0.3960	0.6067	0
cyclohexane	C	0.3905	0.4937	0
*n*-hexane	C (CH_3_)	0.3905	0.7322	0
	C (CH_2_)	0.3905	0.4937	0
water	O	0.3151	0.6364	–0.8340
	H	0.0000	0.0000	0.4170
urea	C	0.3770	0.4170	0.9210
	N	0.3110	0.5000	–0.6930
	O	0.3100	0.5600	–0.6750
	H	0.1580	0.0880	0.2850
TMAO	C	0.3041	0.2826	–0.260
	N	0.2926	0.8360	0.440
	O	0.3266	0.6379	–0.650
	H	0.1775	0.0773	0.110

In many previously reported simulations on similar systems, direct analysis of hydrophobic effects based on two model hydrophobic centers^19,44–50^ or small clusters^51^ was carried out. However, the issue of aggregation-dependent cluster size has already been emphasized,^52,53^ suggesting a favorable attraction only for hydrophobic clusters larger than five moieties, thereby questioning the reliability of those studies. Likewise, this legitimates our choice of using ten hydrophobic solutes.

After a preliminary steepest descent minimization, one round of *NPT* equilibration with position restrains was performed for 10 ns using the Parrinello–Rahman pressure coupling (τ_*P*_ = 0.5 ps). This run ensures a mechanical equilibration, while the volume is fluctuating. The final box volume corresponding to the pressure of 1.01325 bar is then stabilized at the end of the simulation and used in the forthcoming runs. Thereafter, while still keeping the solute’s atoms frozen, we performed a short *NVT* equilibration for 10 ns using the velocity rescaling thermostat (τ_T_ = 0.1 ps), thereby maintaining the temperature around 298.15 K. Finally, fully unrestrained MD runs in canonical *NVT* were performed for 350 ns, and the frames were saved every 25 ps. In all the simulations, a time step of 10^–15^ s was employed, while Newton’s equations of motion were sampled with the leapfrog algorithm.

The nonbonded interactions between atomic sites of two different molecules were modeled as described in eq 1:

1 $$U_{ij}\left(r_{ij}\right)=4\epsilon_{ij}\left[{\left(\frac{\sigma_{ij}}{r_{ij}}\right)}^{12}-{\left(\frac{\sigma_{ij}}{r_{ij}}\right)}^{6}\right]+\frac{q_{i}q_{j}}{r_{ij}}$$

where *q*_*i*_ and *q*_*j*_ are the partial charges on pairwise atoms *i* and *j* separated by the distance *r*_*ij*_, σ_*ij*_ is the distance at which the Lennard-Jones potential is zero, and ϵ_*ij*_ is the well depth. The Lennard-Jones parameters σ_*ij*_ and ϵ_*ij*_ for two interacting sites *i* and *j* were obtained by employing the Lorentz–Berthelot combining rules σ_*ij*_ = (σ_*i*_ + σ_*j*_)/2 and $\epsilon_{ij}=\sqrt{\epsilon_{i}\epsilon_{j}}$ . Moreover, long-range electrostatic interactions were computed with the particle mesh Ewald scheme, while short-range electrostatic and van der Waals interactions were truncated with a single-range cutoff at 12.2 Å with the pair list updated every 10 steps.

The pair radial distribution functions *g*(*r*) were computed for each of the molecular pairs involved, i.e., C_5_H_12_–C_5_H_12_, cC_6_H_12_–cC_6_H_12_, and *n*C_6_H_14_–*n*C_6_H_14_. This enables us to compute the corresponding PMF, *W*(*r*), using the relation:

2 $$W\left(r\right)=-k_{B}T\ln g\left(r\right)$$

where *k*_*B*_ *T*≈ 2.479 KJ mol^–1^ at *T* = 2 98.15 K.

It should however be noted that for the computation of *g*(*r*), we used the central carbon atom of neopentane as reference and the center-of-mass (COM) of both cyclohexane and *n*-hexane, unlike in our previous study, where full atomic positions were employed to compute the PMF.^21^

In order to obtain the enthalpy Δ*H*(*r*) and entropy Δ*S*(*r*) contributions to the PMFs (Δ*G*(*r*), from now on) as a function of solute–solute separation distance *r*, we used the so-called ″finite-difference″ approximation based on the following relation:^44,46^

3 $$\Delta S\left(r\right)=-{\left(\frac{\partial \Delta G\left(r,T\right)}{\partial T}\right)}_{V,N}$$

so that Δ*S*(*r*) can be computed from free energy simulations at different temperatures as follows:

4 $$\Delta S\left(r\right)=-\frac{\Delta G\left(r,T+\Delta T\right)-\Delta G\left(r,T-\Delta T\right)}{2\Delta T}$$

where the thermodynamic quantities are written explicitly as functions of the temperature *T*, and the difference is taken at constant volume (*V*) and particle number (*N*), justifying the uses of canonical *NVT* simulations in this work. The enthalpy Δ*H*(*r*) at the temperature of interest *T* is finally computed as

5 ΔH(r)=ΔG(r)+TΔS(r)

Three simulations at the temperatures *T* and *T* ± Δ*T* are therefore necessary to compute Δ*G*(*r*), Δ*S* (*r*), and Δ*H*(*r*). In our case, the PMFs were computed at 273.15, 298.15, and 323.15 K so that *T* = 298.15 K and Δ*T* = 25 K. This approach assumes that the heat capacity Δ*C*_*v*_ does not change in the temperature range considered:

6 $$\Delta C_{v}={\left(\frac{\partial \Delta S}{\partial T}\right)}_{V,N}\approx constant$$

The standard deviation on Δ*G*(*r*), Δ*S*(*r*), and Δ*H*(*r*) at 298.15 K was evaluated using error block analysis. Each simulation was divided into five individual pools, and standard deviations were computed at each time frame of the trajectories.

### 2.2 Solvation Free Energy

The solvation free energy Δ*G*_*s*_ can be defined as the difference between the free energy of a solute in a specified solvent *G*_*s*_ and in a vacuum *G*_0_:

7 $$\Delta G_{s}=G_{s}-G_{0}$$

If Δ*G*_s_ < 0, the process is spontaneous, indicating that solvation is favored. This concept can clearly be extended to the free energy transfer from solvent *s*_1_ to solvent *s*_2_:

8 $$\Delta \Delta G_{s_{1}>s_{2}}=\Delta G_{s_{2}}-\Delta G_{s_{1}}$$

From the numerical viewpoint, free energy differences can be conveniently computed by using thermodynamic integration:^54^

9 $$\Delta G_{s}=\int_{0}^{1}d\lambda {\langle \frac{\partial V\left(r;\lambda \right)}{\partial \lambda }\rangle }_{\lambda }$$

where *V*(**r**,λ) is the potential energy of the system as a function of the coordinate vector ***r***, and 0 ≤ λ ≤ 1 is a switching-on parameter allowing a gradual change from state λ = 0, where the solute is fully interacting with the solvent, to state λ = 1 where it does not interact at all. The average ⟨···⟩_λ_ in eq. 9 is the usual thermal average with the potential *V*(**r**,λ) = (1−λ)*V*(**r**,0) + λ*V*(**r**,1) at a fixed value of λ. The λ interval [0,1] is partitioned into a grid of small intervals, molecular dynamics simulations are performed for each value of λ belonging to each interval, and the results are then integrated over all values of λ to obtain the final free energy difference. In the present study, 21 lambda points for each simulated system were used.

The solvation free energy was computed for each of the three hydrophobic solutes, neopentane, cyclohexane, and *n*-hexane, in water at 298.15 K using a single solute following our previous protocol.^20–22^ It is worth noting that simulations were performed with the GROMOS96 (54a7) force-field^55^ with SPC/E water model,^56^ and both solutes were modeled in their united atom conformations. A simulation time step of 1 fs was used. The accurate leapfrog stochastic dynamics integrator was applied in all the simulations, with the Parrinello–Rahman coupling barostat. Performing this calculation at different temperatures allows to single out the individual contributions of the solvation enthalpy Δ*H*_s_ and entropy Δ*S*_s_ as in refs. 20–22

### 2.3 β2m MD Simulations

#### 2.3.1 Atomistic Molecular Models

In order to provide a more factual and general perspective to the simplistic hydrophobic simulation models comprising neopentane C_5_H_12_, cyclohexane cC_6_H_12_, and *n*-hexane *n*C_6_H_14_, simulations were also performed on realistic-like hydrophobic and suited paradigmatic protein models, β2m, in the same conditions as described for hydrocarbons above, see system description depicted in Figure 1.

The starting structure was obtained by excising the X-ray coordinates of β2m (chain B) that is part of the major human histocompatibility antigen HLA-A2 complex solved at 2.6 Å resolution (PDB ID: 3HLA).^30^ All the external crystallographic water solvent was removed, and missing hydrogens were added using the *pdb2gmx* utility of the Gromacs software package. β2m was placed at the center of a cubic box (512000 Å^3^) at a minimum distance of 15 Å from the edges and solvate by the rigid 3-site TIP3P water^37^ molecules. The molecular interactions were accounted for using the amber99sb-ildn force field.^57^ Subsequently, one counterion was added to achieve eletroneutrality. We should, however, stress that a sufficiently large box (unit box length 80 Å) is used to mitigate the finite box effects. Cosolvents urea and TMAO were added to an aqueous solution with the same concentration as in the hydrophobic representative models. The full system’s composition is shown in Table 1.

#### 2.3.2 All-Atom Simulation Details

The solute’s potential energy of the solvated systems was minimized by relaxing the solvent around the solute atoms before running the unrestrained MD simulations. During the energy minimization stage, we employed the steepest descent minimization algorithm with a minimization step size of 0.01 nm and a maximum convergence force of 500.0 kJ mol^–1^nm^–1^. Thereafter, an equilibration round in the canonical *NVT* ensemble was performed for 5 ns using the leapfrog integrator with a simulation time step of 1 fs. While long-range electrostatics interactions were accounted for with the particle mesh Ewald summation, short-range electrostatics and van der Waals interactions were truncated with a single-range cutoff at 12 Å with the pair list updated every 20 steps. The velocity for the Maxwell distribution temperature was set to 300 K. The temperature of the full system was equilibrated to this latter reference value using the velocity rescaling (modified Berendsen thermostat)^58^ with a coupling constant of 1.0 ps. To mimic the density of the realistic bulk-like phase, all the simulations were replicated in the 3D space using periodic boundary conditions, and all bonds involving hydrogen atoms were restrained using LINCS algorithms.^59^

The second equilibration run lasts 5 ns and was performed in the isobaric–isothermal *NPT* ensemble using the same parameters as those described above for *NVT*. Moreover, the pressure was kept around the reference value of 1 bar using the Parrinello–Rahman pressure coupling^60^ with a coupling constant of 2.0 ps. In the final production stage, the restraints on heavy atoms were released, and the systems evolved for 200 ns using a integration time step of 2 fs without imposing any constraints on solute’s bending and dihedral degrees of freedom (see Table 1).

### 2.4 Preferential Binding Coefficients, Γ

The addition of small molecules (cosolvents or osmolytes) to the aqueous protein solution likely leads to the perturbation of its local solvation environment, potentially perturbing its chemical potential. These small molecules may more strongly (destabilize) or weakly (stabilize) interact with protein than water, the so-called ″preferential interaction″.^10^ Indeed, this is nothing but the measure of the excess number of water or cosolvent molecules in the local domain of the protein surface. The computation of the preferential interaction constant, Γ, requires no prior knowledge of the binding site and can be estimated directly from MD simulation following the equation:^13^

10 $$\Gamma =\left\langle n_{\gamma }\left(r\right)-\frac{n_{\gamma }^{\mathrm{tot}}-n_{\gamma }\left(r\right)}{n_{w}^{\mathrm{tot}}-n_{w}\left(r\right)}\times n_{w}\left(r\right)\right\rangle$$

where *n*_γ_ (*r*) and *n*_*w*_(*r*) are the number of cosolvents (urea or TMAO) and water molecules at the cutoff distance *r* from the hydrophobic solute or protein surface. Superscripts ″tot″ denote the total number of cosolvents or water molecules in the system. The average ⟨···⟩ represents the thermal averaging. The distance-dependent Γ plot enables to identify the appropriate value of the cutoff distance *r* needed to unequivocally estimate the value of Γ. This marks the boundary between the local and bulk domains of the solvated system.

In general, denaturants exhibit a positive (destabilizing) value of Γ, pointing to an accumulation of the cosolvent in the local shell of the protein owing to a net favorable interaction.^12^ Meanwhile, protecting osmolytes display negative (stabilizing) values of Γ pertaining to their exclusion from the local domain of the protein as a result of net unfavorable interactions with the protein surface.^12^

## 3 Results and Discussion

### 3.1 Solute–Solute Pair Radial Distribution Functions in Water and Aqueous Osmolyte Mixtures

The aggregation propensity mimicking the local structural arrangement of the different hydrophobic models studied in this work, propelled by the presence of cosolvents, can be characterized by means of intermolecular solute–solute pair radial distribution functions. This analysis was conducted using the central carbon atom of neopentane C_5_H_12_ as reference, while the COMs of cyclohexane cC_6_H_12_ and *n*-hexane *n*C_6_H_14_ were considered, given rise to the plots shown in Figure 2. However, before jumping into that, it proves instructive to have a look at the convergence trend of the simulations trajectories under the conditions applied here, namely, constant temperature (for hydrophobic solute models) and pressure (for β2m runs), as reported in Table SI, Figures SI, and SII. It is clear that both temperature and pressure have reached reasonable equilibrium in the course of the simulations as witnessed by the steady pattern over the time-dependent fluctuations and small deviation from average (±2 K) in the case of temperatures. Nonetheless, standard deviation on pressure is noteworthy, in the range of ± 180 bar, consistent with its known large fluctuation propensity.^61^ Remarkably, it appears that urea most likely lowers the average pressure of the system at the opposite of TMAO which rises it, a landmark evidence of their counteracting effects on protein stability, further confirmed when both entities are present in a solution with a steady restoring of the average pressure around 1 bar as in the pure water system (see Table SI).

**Figure 2:**
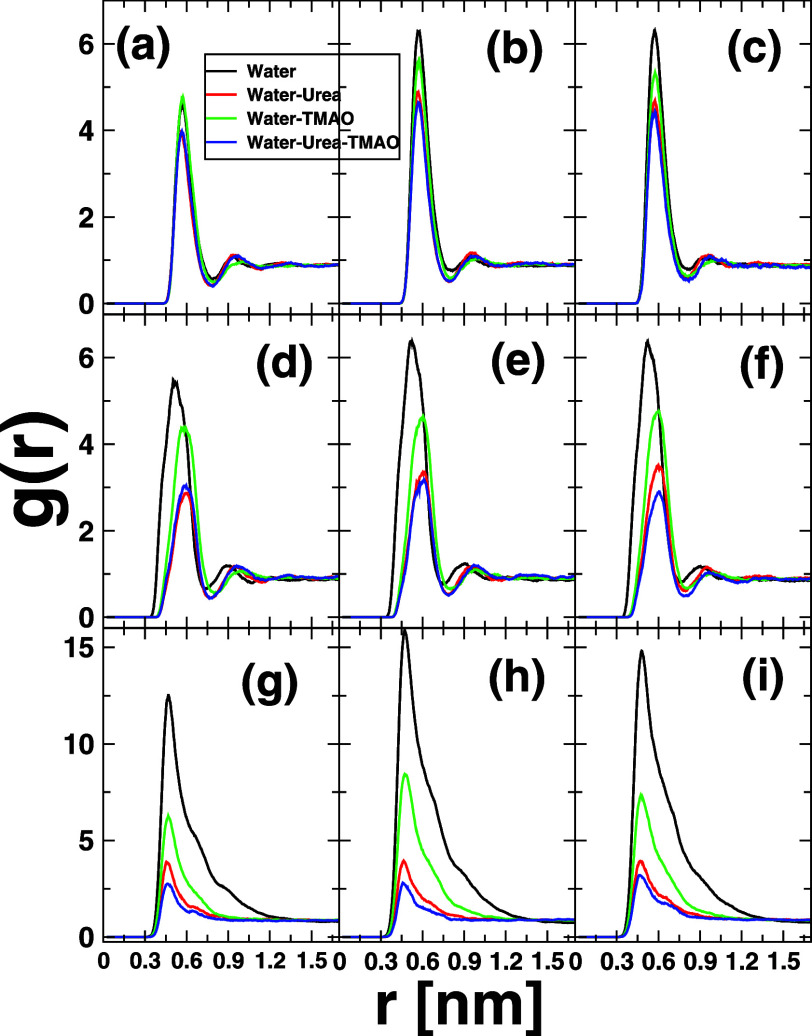
Solute–solute pair radial distribution functions for each of the hydrocarbon solutes considered herein at different temperatures. The top panel (a–c) displays the results of neopentane C_5_H_12_, the central panel those of cyclohexane cC_6_H_12_ (d–f), and the bottom panel the *n*-hexane *n*C_6_H_14_ (g–i) counterparts, respectively. From left to right, the results obtained at different temperatures are shown: 273.15 K (left), 298.15 K (middle), and 323.15 K (right). Results obtained in pure water are plotted in black lines, those referring to 8.16 M water-urea solution in red, while green lines refer to data in 3.48 M water-TMAO binary mixture, and blue lines showcase the results in water-7.18 M urea-2.87 M TMAO ternary mixture. The central carbon atom of neopentane was used to compute the RDFs, while the COM was considered for cyclohexane and *n*-hexane. Please note that *n*-hexane plots are not on the same scale as those of neopentane and cyclohexane.

Figure 2 emphasizes the inter solute *g*(*r*) for each of the hydrocarbon models studied here at different temperatures. From left to right, the data obtained at 273.15 K (a–c), 298.15 K (d–f), and 323.15 K (g–i) are, respectively, displayed. From top to bottom, the panels correspond to the results of different solutes: neopentane C_5_H_12_ (top), cyclohexane cC_6_H_12_ (middle), and *n*-hexane *n*C_6_H_14_ (bottom). The color code reads as black (pure water), red (8.16 M water-urea), green (3.48 M water-TMAO), and blue (water-7.18 M urea-2.87 M TMAO). The main highlights from this analysis are as follows. In general and independently to the solute model considered, the first peak height is found to be highest in the case of pure water H_2_O, somehow pointing to the fact that water is a poor solvent for hydrophobic collapse^21,22^ and, in this particular case, drives out more strongly the hydrophobic association compared to the binary and ternary cosolvent phases. Moreover, the peak height that should indeed correlate with the strength of hydrophobic association as described later on in Figure 3 decreases in the order: water > water-TMAO > water-urea > water-urea-TMAO. This clearly evidenced nothing, but the assertion is cosolvents urea and TMAO alter the structure of the water network, thereby weakening the strength of hydrophobic association. Besides, Figure 2 reports only a relatively small temperature dependence of peak height and position, at least in the temperature range considered here. On the system-dependent base, it is noteworthy that neopentane C_5_H_12_ and cyclohexane cC_6_H_12_ exhibit a clear second coordination shell, which is not the case for *n*-hexane *n*C_6_H_14_. In addition, the latter system displays the highest height first coordination peak compared to the two others, likely ascribed to its slightly more hydrophobic character than C_5_H_12_ and cC_6_H_12_, and most probably also to its extended shape which may promote more *n*C_6_H_14_–*n*C_6_H_14_ contacts and thus hydrophobic paring. While the trend in solute–solute hydrophobic association is rather clear from pair radial distribution functions in Figure 2, the strength and thermodynamics underlining this aggregation-like process can only be unveiled by computing the corresponding PMFs.

**Figure 3:**
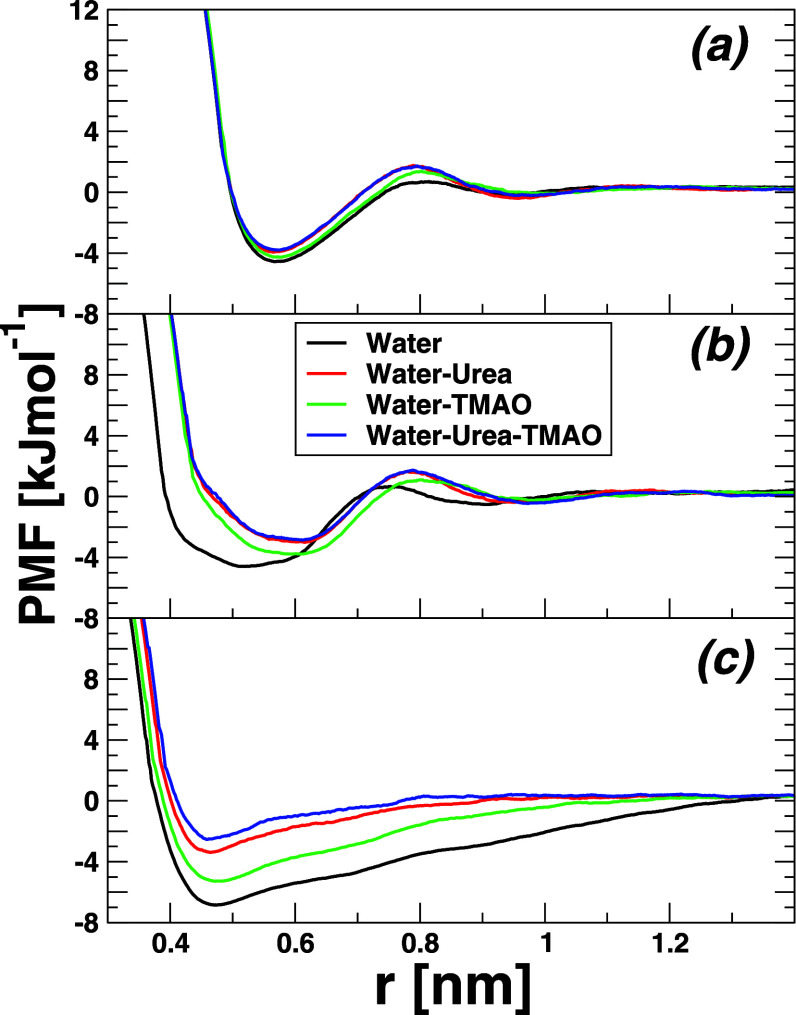
Potentials of mean force at 298.15 K of neopentane C_5_H_12_ (a), cyclohexane cC_6_H_12_ (b), and of *n*-hexane *n*C_6_H_14_ (c). Molecules in water (black), 8.16 M water-urea (red), 3.48 M water-TMAO (green), and water-7.18 M urea-2.87 M TMAO ternary mixture (blue). The considered moieties are the central atom of neopentane C_5_H_12_ and the center of mass of both cyclohexane cC_6_H_12_ and *n*-hexane *n*C_6_H_14_.

### 3.2 Potential of Mean Force of Neopentane C_5_H_12_, Cyclohexane cC_6_H_12_, and *n*-Hexane *n*C_6_H_14_ in Water and Aqueous Osmolyte Mixtures

A cornerstone of many fundamental processes in biology and chemistry is the potential of mean force (PMF) between nonpolar moieties in water, a prototypical way to mimic hydrophobic effects. The PMF puts into words the energetics involved when hydrophobic solutes in a mutual solvent aggregate to avoid unfavorable interactions with water,^62^ thus embedding desolvation and binding. In Figure 3, we have reported the solute–solute PMFs in the various solvent compositions investigated herein. It appears that, as noticed before, for the association of small molecules of methane or LJ solutes,^8,63,64^ the PMF curves present multiple minima as a function of solute separation distance. In particular, the PMFs of neopentane C_5_H_12_ (Figure 3a) and cyclohexane cC_6_H_12_ (Figure 3b) show similar features, with two minima separated by a maximum corresponding to the desolvation barrier (DSP) around 0.8 nm. The first and deepest minimum, also known as the contact minimum (CM), showcases the direct interaction among hydrophobic solutes and is found to be around 0.6 nm in both cases. The second minimum, located around 1.0 nm (large enough to host water molecules between solute dimers), is the signature of solvent-separated (SSM) hydrophobic pairs. However, the case of *n*-hexane nC_6_H_14_ appears to be more peculiar (see Figure 3c). Indeed, the corresponding PMF exhibits a well-localized contact minimum, slightly shifted inward at ca. 0.47 nm, with no clear desolvation peak or solvent-separated minimum. Instead, a seemingly asymptotic-dependent behavior is reported, more likely resembling the PMF of hydrophobic polymers in water.^65^ Clearly, this result certainly agrees with the PMF system size-dependent feature already reported before and portrays the difference in geometry, cyclic versus linear shapes of cyclohexane and *n*-hexane, respectively. Interestingly, Figure 3 clearly shows a noticeable increase of the DSP alongside with the decrease of the well depth at the CM upon addition of osmolytes urea and TMAO. The PMF values at the CM, DSP, and SSM are summarized in Table 3.

**Table 3:** Result Statistics of PMFs (in kJmol^–1^) for Different Solute–Solute Configurations including at the Contact Minimum (CM), the Desolvation Peak (DSP), and Solvent-Separated Minimum (SSM)

Systems	Cosolvents	CM (kJmol^–1^)	DSP (kJmol^–1^)	SSM (kJmol^–1^)
neopentane	water	–4.536	0.701	–0.169
	water-urea	–3.925	1.756	–0.391
	water-TMAO	–4.268	1.369	–0.072
	water-urea-TMAO	–3.792	1.727	–0.212
cyclohexane	water	–4.589	0.669	–0.523
	water-urea	–3.000	1.654	–0.355
	water-TMAO	–3.783	1.098	–0.235
	water-urea-TMAO	–2.858	1.740	–0.457
*n*-hexane	water	–6.846	–4.990	–3.202
	water-urea	–3.391	–1.190	–0.043
	water-TMAO	–5.281	–3.095	–1.100
	water-urea-TMAO	–2.540	–0.442	0.240

Except for the case of *n*-hexane, the results reported in Figure 3 and Table 3 all agree that the PMF at the CM is deeper in pure water than in osmolyte mixture solutions. Likewise, the height of the DSP and the depth of the SSM are smaller (more negative) in a pure water solution than in the other solvents. This consistently supports nothing but the higher proficiency of hydrophobic entities to collapse and form aggregates in pure water than in a water-osmolyte multiphase solution. Moreover, the positions of the CM and DSP are only slightly affected by the addition of osmolyte compared to that of SSM, wherein one witness a shift outward to larger solute distances, and noteworthy in the case of cyclohexane cC_6_H_12_. In short, upon the addition of osmolyte, the depth of the CM decreases and the height of the DSP increases, somehow implying a less hydrophobic propensity than pure water. The trend observed here is in accord with the previously reported data.^6,7,48^ We should however notice that, albeit qualitatively and quantitatively similar to the earlier results by Sarma and Paul^7^ for neopentane C_5_H_12_, some discrepancies can be traced back between our results and those reported by Lee and van der Vegt^19^ and van der Vegt et al.^44^ for neopentane C_5_H_12_ in water and water-urea, notably as far as the depth of the hydrophobic attraction is concerned. We surmise that this is a signature of the PMF-dependent water model, TIP3P^37^ here and SPC/E^56^ in the case of van der Vegt et al. study.^44^

In the realm of protein (un)­folding dynamics, CM and SSM pertain roughly to the favorable hydrophobic contacts in the (un)­folded state of the protein and thus correlate well to their corresponding activation barriers. To mimic this effect, we have reported in Table 4 the relative changes of the desolvation barrier with respect to the CM (Δ*G*_*u*_) and SSM (Δ*G*_*f*_) and the free energy difference between SSM and CM (Δ*G*_f→u_). From Table 4, it is unambiguously seen that for all the hydrophobic systems understudied, the addition of osmolyte leads to the enhancement of the (un)­folding activation barrier. Thus, CM and SSM configurations are favored upon the addition of osmolytes. This relative osmolyte-induced stabilization of the SSM contacts is linked to the increased solubility of the hydrophobic solutes considered here in osmolyte solutions compared to pure water. Indeed, the computed solvation free energy for neopentane C_5_H_12_ at 300 K changes as follows: Δ*G*_solv_(water-urea-TMAO) < Δ*G*_solv_(water-urea) < Δ*G*_solv_(water-TMAO) < Δ*G*_solv_(water). Neopentane is mentioned just as a case illustration, the same trend being observed also for other hydrophobic solutes. Furthermore, temperature dependence solvation free energy for each of the hydrocarbons considered here in water H_2_O is shown in Figure SIII. This part related to solvation free energy in different solvent compositions will be further detailed somewhere else.

**Table 4:** PMF Differences (in kJmol^–1^) between the Desolvation Peak and Contact Minimum (Δ*G*_u_), Solvent-Separated Minimum and Desolvation Peak (Δ*G*_f_), and Solvent-Separated Minimum and Contact Minimum (Δ*G*_f→u_)

Systems	Cosolvents	Δ*G*_u_ (kJmol^–1^)	Δ*G*_f_ (kJmol^–1^)	Δ*G*_f→u_ (kJmol^–1^)
neopentane	water	5.237	–0.870	4.367
	water-urea	5.681	–2.147	3.534
	water-TMAO	5.637	–1.441	4.196
	water-urea-TMAO	5.519	–1.939	3.580
cyclohexane	water	5.258	–1.192	4.066
	water-urea	4.654	–2.009	2.645
	water-TMAO	4.881	–1.333	3.548
	water-urea-TMAO	4.598	–2.197	2.401
*n*-hexane	water	1.856	1.788	3.644
	water-urea	2.201	1.147	1.147
	water-TMAO	2.186	1.995	4.181
	water-urea-TMAO	2.098	0.682	2.780

### 3.3 Temperature Dependence of PMFs

The stability of the CM, DSP, and SSM configurations depends on many factors among which the concentration of the cosolvents,^48^ the forcefield model used,^48^ and the temperature.^44,48,50,66^ In particular, knowledge of the temperature dependence of the hydrophobic association is of paramount importance since it enables us to single out the contributions of enthalpy and entropy to the PMFs and thus provides a complete description of the solvation thermodynamics. Figure SIV reports the solute pair PMFs in pure water H_2_O and aqueous osmolytes urea and TMAO at three temperatures 273.15, 298.15, and 323.15 K. The left panel shows the neopentane C_5_H_12_ results (a–d), the central panel shows those of cyclohexane cC_6_H_12_ (e–h), and the rightmost panel reports on the results of *n*-hexane *n*C_6_H_14_ (i–l). From top to bottom, the panels show the results obtained in pure water, 8.16 M water-urea, 3.48 M water-TMAO, and water-7.18 M urea-2.87 M TMAO ternary mixture, respectively. The black line refers to the temperature of 273.15 K, the red to 298.15 K, and the blue one to 323.15 K. Similar to the plot reported in Figure 3, the positions of CM, DSP, and SSM kept relatively unchanged. More precisely, in general, the contact minimum CM is shifted slightly to larger distances and is noteworthy in the case of neopentane C_5_H_12_. Concomitantly, the solvent separated minimum becomes more shallow and even collapses in all the systems considered here. Besides, we can notice that the first PMF peak (CM) becomes deeper upon temperature increase independently to the solute and/or solvent considered. This is a landmark fingerprint of an entropic association scheme, as noticed before.^44,48^ A wealth of precious insights into the hydrophobic association mechanisms can be obtained from the temperature dependence of the PMFs, and more in particular, we can single out the thermal components of hydrophobic interactions, i.e., enthalpy and entropy, as reported in Figure 4.

**Figure 4:**
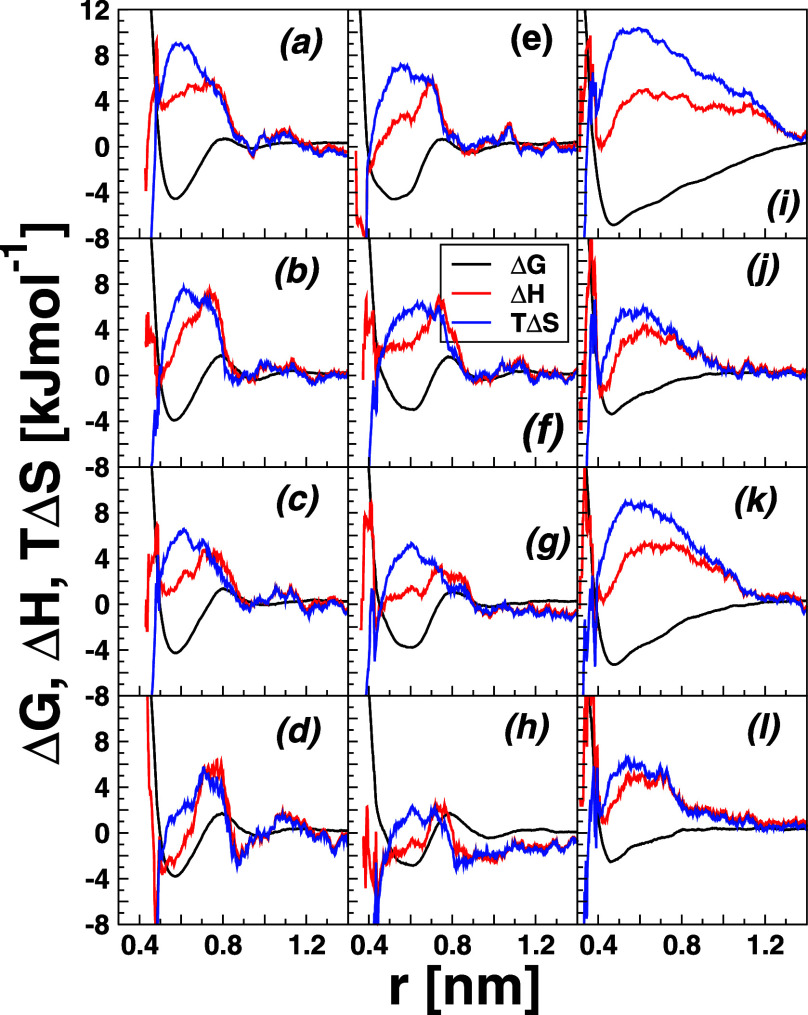
Enthalpy and entropy contributions to the potential of mean force at 298.15 K of neopentane C_5_H_12_ (a–d) (left), cyclohexane cC_6_H_12_ (e–h) (middle), and of *n*-hexane *n*C_6_H_14_ (i–l) (right) molecules. From top to bottom, panels show the results in water solution, 8.16 M water-urea, 3.48 M water-TMAO, and water-7.18 M urea-2.87 M TMAO ternary mixture, respectively. The black line refers to the free energy (PMF), the red line to enthalpy, and the blue one to entropy.

### 3.4 Enthalpic and Entropic Contributions to PMFs

Figure 4 reports the enthalpy and entropy contributions to the PMFs as a function of the intersolute separation distance at 298.15 K of neopentane C_5_H_12_ (a–d) (left panel), cyclohexane cC_6_H_12_ (e–h) (central panel), and *n*-hexane *n*C_6_H_14_ (i–l) (right panel). From top to bottom, the results obtained in pure water, 8.16 M water-urea, 3.48 M water-TMAO, and water-7.18 M urea-2.87 M TMAO ternary mixture are, respectively, displayed. The black line refers to the PMFs (Δ*G*), the red to enthalpy (Δ*H*), and the blue to entropy (Δ*S*). It is seen from Figure 4 that, in all the solute and solvent studied, enthalpy and entropy have opposite signs (Δ*H* < 0 and *T*Δ*S* > 0) at the CM, though, with a dominant entropic contribution. Furthermore, there is a reasonable trend of enthalpy and entropy to fluctuate in phase with fairly analogous amplitudes, a signature that both contributions should reasonably cancel out in the free energy (PMF = Δ*H* – *T*Δ*S*) at the CM, and noteworthy in the case of *n*-hexane *n*C_6_H_14_. More specifically, in all the cases presented in Figure 4, it is clear that, although the CM configurations are largely entropically driven (consistent with refs. 44,46,49,67,68 ), both entropy and enthalpy favored the solute–solute configurations at the CM.^68^ Noteworthy, compared to pure water, the enthalpic and entropic contributions to PMFs decrease at favorable solute–solute contacts at the CM.

As far as the SSM contacts are concerned, both contributions are nearly isoweighted, mimicking a perfect enthalpy–entropy compensation scheme at SSM configurations, in accord with the previous results of van der Vegt et al.^44^ In addition, albeit exhibiting some variegated features throughout the different subplots of Figure 4, SSM configurations are dominantly enthalpically driven, induced by water hydrogen bonding.^49,68^ Specifically, in the case of binary water-urea and ternary water-urea-TMAO solutions, TMAO preferentially interacts with the hydrocarbons (C_5_H_12_, cC_6_H_12_, and *n*C_6_H_14_) over water H_2_O (see Figure SV), thereby promoting more water–water contacts, and thus hydrogen bonding, thereby leading to a favorable enthalpic contribution at the SSM. Regarding the hydrophobic contacts at the DSP, Figure 4 depicts that those configurations are stabilized by entropy and opposed by enthalpy, owing to the lower propensity of hydrogen bond formation.

The calculation of entropy from numerical differentiation shown in Figure 4 appears to display noticeable fluctuations. Thus, it proves relevant to compute the errors on this particular observable as well as on enthalpy and free energy. This was done using error block analysis as described in Section 2, and the results are shown in Figures SVI, SVII and SVIII for Δ*G*(*r*), Δ*H*(*r*), and Δ*S*(*r*) at 298.15 K, respectively. In the case of free energy Δ*G*, the errors shown in Figure SV appear to fall within about 15% of the average, thus highlighting a pretty well fitting of free energy Δ*G* all over the different subset systems investigated and specifically at the CM, SSM, and DSP contacts. Conversely, numerical fluctuations are amplified on entropy Δ*S* in Figure SVII and eventually more on enthalpy Δ*H* in Figure SVIII. This is somehow congruent with the finite-difference derivation used here which indeed turns up errors on entropy (see eq 4) relative to free energy, with an even more significant error component on enthalpy as a result of the summation over Δ*G* and Δ*S* terms (see eq. 5). Evidently, achieving convergence on entropy and enthalpy relative to free energy is always a bottleneck issue, despite the sampling achieved here, 350 ns. Thus, longer sampling may be needed to mitigate the hard core issue of achieving convergence on both entropy and enthalpy relative to free energy and especially from numerical derivations such as the finite-difference approximation employed here.

### 3.5 Preferential Interaction Coefficient

The preferential solvation is the thermodynamics observable used to estimate the excess number of cosolvent entities surrounding a specific solute compared to that of the bulk. Thus, it is a proxy to measure the deviation from an ideal solvation model. It was computed using eq. 10 as described in Section 2. Figure SV reports the distance-dependent preferential interaction coefficients of neopentane C_5_H_12_, cyclohexane cC_6_H_12_, and *n*-hexane *n*C_6_H_14_. The vertical broken line marks the boundary between the local and bulk domains and indicates the cutoff distance used to estimate the value of Γ shown in Table 5.

**Table 5:** Preferential Interaction Coefficients Γ for Each of the Multiphase Systems Investigated Here at 298.15 K; in the Case of the Water-Urea-TMAO System, the First Line Refers to Urea, While the Second One Points to TMAO

Systems	Cosolvents	Γ
neopentane	water-urea	1.59 ± 0.08
	water-TMAO	0.76 ± 0.03
	water-urea-TMAO	0.60 ± 0.06
		0.31 ± 0.02
cyclohexane	water-urea	2.36 ± 0.08
	water-TMAO	0.90 ± 0.03
	water-urea-TMAO	0.58 ± 0.24
		0.46 ± 0.11
*n*-hexane	water-urea	2.22 ± 0.13
	water-TMAO	1.26 ± 0.09
	water-urea-TMAO	1.59 ± 0.07
		0.43 ± 0.05
β2m	water-urea	26.11 ± 3.82
	water-TMAO	-7.40 ± 0.69
	water-urea-TMAO	43.39 ± 5.66
		-19.19 ± 4.03

Both hydrophobic solutes considered herein display a similar trend. In Figure SV, neopentane C_5_H_12_ (Figure SVa) preferentially interacts with both urea and TMAO (Γ > 0) except from the close vicinity of the solute within about 5.5 Å (Γ < 0). However, the strength of interaction is weaker in TMAO than in urea, as witnessed by the small Γ value of the former than in the later (see Table 5). The same analysis can accurately be done for cyclohexane cC_6_H_12_ (Figure SVb) and *n*-hexane *n*C_6_H_14_ (Figure SVc). In particular, in their inner solvation shells, albeit both of them likely interact with urea and TMAO over water H_2_O (Γ > 0), a strong preference is given to urea moieties. Besides, unlike the case of neopentane C_5_H_12_, in the ternary water-urea-TMAO solution, TMAO actions’ seem not to preclude urea’s accumulation to the hydrocarbon surfaces. Altogether, the data presented in Figure SV support nothing but the hydrophobic solutes dehydration induced by osmolytes, owing to their preferential binding with these latter.^7^ This analysis is further supported by the direct number of interacting cosolvents around the first solvation shell of the hydrophobic solutes, as displayed in Table 6. Indeed, overall, Table 6 points out that the number of interacting urea moieties within the first solvation shell of all of the hydrophobic systems studied is definitely larger than the TMAO counterparts.

**Table 6:** Solute–Solute and Solute–Solvent Running Coordination Numbers around the First Solvation Shell for Each System Studied Here at 298.15 K^a^ ^b^ c

Systems	Cosolvents	*n*_s_	*n*_u_	*n*_t_	*n*_w_
neopentane	water	0.37	–//–	–//–	30.41
	water-urea	0.22	7.01	–//–	14.62
	water-TMAO	0.28	–//–	3.63	19.29
	water-urea-TMAO	0.18	5.75	2.64	12.04
cyclohexane	water	0.41	–//–	–//–	14.51
	water-urea	0.19	7.28	–//–	8.40
	water-TMAO	0.30	–//–	3.92	10.77
	water-urea-TMAO	0.16	6.34	2.74	6.37
*n*-hexane	water	1.73	–//–	–//–	16.23
	water-urea	0.36	6.48	–//–	9.93
	water-TMAO	0.72	–//–	5.04	13.76
	water-urea-TMAO	0.25	5.92	2.31	8.77
β2m	water	–//–	–//–	–//–	77.91
	water-urea	–//–	145.17	–//–	56.81
	water-TMAO	–//–	–//–	50.94	72.77
	water-urea-TMAO	–//–	147.55	39.72	49.32

a *n* is the number of solutes or cosolvents around each simulated solute with subscripts s, u, t, and w referring to solute (neopentane or cyclohexane or *n*-hexane or β2m), urea, TMAO, and water, respectively.

b The first shell corresponds to the first minimum in the pair radial distribution function.

c In the case of *n*-hexane, there is not clear localized first minimum in the *n*-hexane–*n*-hexane radial distribution function. Therefore, the cutoff used to count the number of neighbors is 1 nm, nearly corresponding to the point at which the plot becomes flat and the curvature changed.

### 3.6 Water–Water Pair Radial Distribution Functions

We computed the water–water pair radial distribution functions (RDFs) in pure water and in aqueous osmolyte mixtures. As a matter of fact, this could enlighten us about the induced effects of cosolvent urea and TMAO on water structure, potentially disclosing insightful details due to the addition of osmolyte on the hydration propensity of the hydrocarbons studied herein. This analysis is reported in Figure[Fig fig5]a for neopentane C_5_H_12_, Figure[Fig fig5]b for cyclohexane cC_6_H_12_, and Figure 5c for *n*-hexane *n*C_6_H_14_. The color code for each line is the same as that in Figure 3.

**Figure 5:**
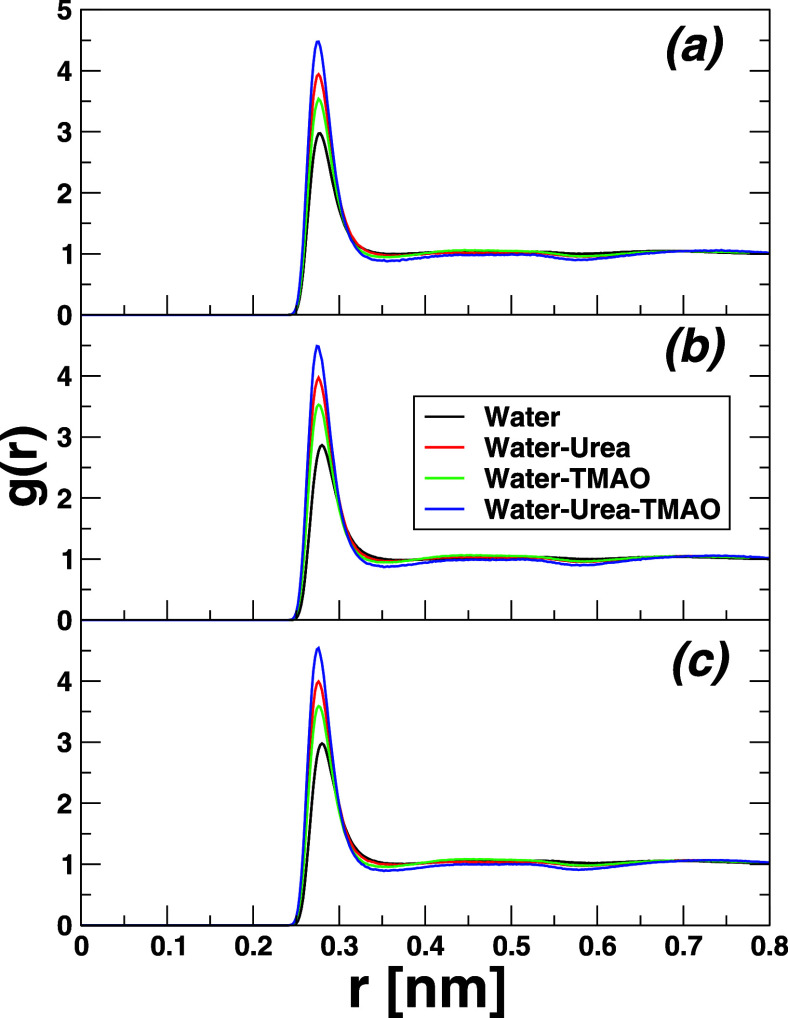
Site–site water oxygen-water oxygen pair radial distribution functions for each simulated systems embedding neopentane C_5_H_12_ (a), cyclohexane cC_6_H_12_ (b), and *n*-hexane *n*C_6_H_14_ (c). The lines represent the simulations in pure water solution (black), 8.16 M water-urea (red), 3.48 M water-TMAO (green), and water-7.18 M urea-2.87 M TMAO ternary mixture (blue), respectively.

In Figure 5, both profiles are strongly conservative independently to the hydrocarbon solute considered and display characteristic behavior akin to water–water correlation functions.^56^ More precisely, the locations of the first and second peaks in Ow-Ow RDF plots at nearly 0.28 and 0.45 nm are fingerprints of the hydrogen-bonded first neighbor and the tetrahedrally located second neighbor, respectively, and in good agreement with previously reported results.^7,69^ Nonetheless, it should be highlighted that the addition of cosolvents urea and TMAO slightly induces the shift of the first peak position inward with respect to pure water. Most importantly, it is evident that both urea and TMAO reasonably enhance the first peak height, with a more pronounced effect due to the addition of urea to the binary water-TMAO solution, i.e., the ternary phase water-urea-TMAO. A plausible hypothesis to this observed change is likely an enhancement-induced water structure due to osmolyte urea and TMAO,^69^ again thrived by their preferential binding with hydrocarbons than water, as shown in Table 5.

In order to scrutinize the orientation of water and cosolvents around the hydrocarbons studied, i.e., neopentane C_5_H_12_, cyclohexane cC_6_H_12_, and *n*-hexane nC_6_H_14_, we computed the RDFs of each of them with each component of the cosolvents including water H_2_O, urea, and TMAO. The results are disclosed in Figure 6, wherein the top panel, Figure 6a–c showcases the results of neopentane C_5_H_12_, the central panel Figure 6d–f those of cyclohexane cC_6_H_12_, and the bottom panel Figure 6g–i the *n*-hexane *n*C_6_H_14_ counterparts. In computing the RDFs, selected molecule sites were employed including the central carbon atom for neopentane, the COMs for cyclohexane and *n*-hexane, all the atoms of water, and oxygen, carbon, and nitrogen atoms for urea and TMAO.

**Figure 6:**
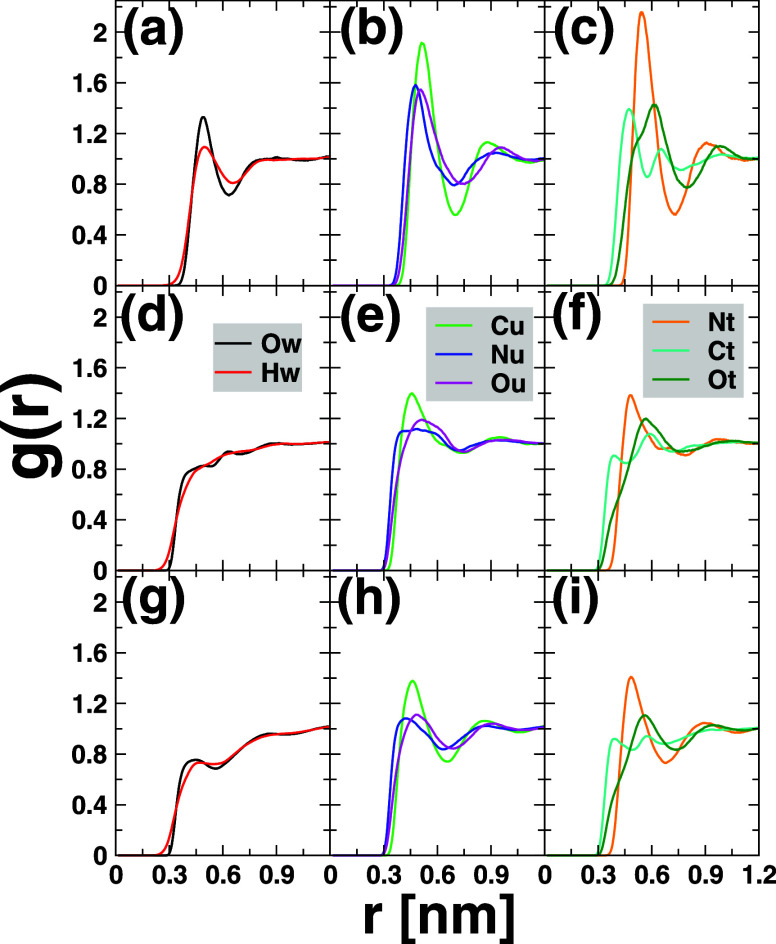
Solute–solvent site–site pair radial distribution functions in different systems studied for the ternary phase water-urea-TMAO. From top to bottom, the results of neopentane C_5_H_12_ (a–c), cyclohexane cC_6_H_12_ (d–f), and *n*-hexane *n*C_6_H_14_ (g–i) are, respectively, displayed. From left to right, the corresponding results for water H_2_O, urea, and TMAO, respectively. While the central carbon atom of neopentane was used to compute the RDFs, the COM was considered for cyclohexane and *n*-hexane. In the case of water, both oxygen and hydrogen atoms were used, whereas for urea and TMAO, all their building atoms were used except hydrogens.

Albeit with a less pronounced propensity in cyclohexane and *n*-hexane than neopentane, the first peak RDFs of both water Ow and Hw atoms surrounding the hydrocarbon solutes lie likely at the same distance, in line with previous results for neopentane.^7^ This is an indication that water moieties lie parallel at the hydrophobic surface, with a smaller tilt deviation angle in the case of cyclohexane and *n*-hexane than in neopentane. In addition, upon moving from neopentane to cyclohexane or *n*-hexane, there is noticeable drift in the first peak intensity, witnessing the decrease in the number of water molecules in the first solvation shell of these latter compared to the former, as also reported in Table 6.

Regarding the case of urea shown in Figure 6b–h, the RDF profiles are qualitatively similar for the three hydrophobes studied here, though with a smaller peak intensity in cyclohexane and *n*-hexane relative to neopentane and a more sharp first peak distribution in the latter than in these first. Specifically, atoms Nu and Ou of urea exhibit similar distribution patterns with two maxima separated by the well. The first and sharp Nu peak likely tells us about the relative tendency of one amino −NH_2_ group of urea to point toward the solute surface, while the broad Ou peak (especially in Figure 6e–h) traduces its affinity to be oriented toward the bulk phase. In short, the data shown in Figure 6 support the sideways alignment of urea moieties near the hydrophobic surface.^7^

For TMAO, it is clear that carbon atoms Ct are closer to the hydrophobic surface in both hydrocarbons considered herein, followed by nitrogen Nt and oxygen atoms Ot. Besides, the broad first and more distant peak distribution of oxygen Ot atoms highlights its potential preference to be oriented toward the bulk solution. Thus, these data fully align with the hypothesis of side-on preference orientation of TMAO units around the hydrophobic surface with the methyl group −CH_3_ pointing toward the surface and the oxygen atom Ot heading for the bulk solution. Interestingly, it is seen that carbon atom Ct distributions further exhibit at least three maxima at larger distance. This is a signature that at least one methyl group −CH_3_ of TMAO is outbound to the hydrophobic surface. This supports nothing but the amphiphilic-like property of TMAO which mediates the formation of hydrogen bonds between the oxygen Ot atom and water and/or urea while still favoring the hydrophobic contacts with the surface, in accord with previous works.^7–9^

The addition of cosolvents into pure water undoubtedly induces the reorganization of the solvation structure around the solute surface, eventually leading to the breaking of the homogeneous isotropic symmetry characteristic of the bulk water phase. To examine the extent to which osmolytes urea and TMAO potentially affect the solvation environment around the solutes, we computed their respective angular distributions in the solute solvation shell using the θ angle defined as in Figure SIX. The results are shown in Figure SX for all of the hydrophobic moieties studied.

The solvation shell of the three solutes studied herein defined by the first peak in their respective radial distribution functions enables counting the number of each cosolvent type around the corresponding solute as shown in Table 6. Figure SX then provides an outlook on the orientation of these molecules around the solutes. As far as the water molecule is concerned, the distributions of *P*(cosθ) plots appear quite consistent among the different solute and solvent mixtures investigated. However, a non-Gaussian-like trend dominates the distributions of θ angles, a firmly indication of a preferred orientation of water entities near the hydrophobic solutes, as already seen in the RDF plots in Figure 6. It is also noticed that the cosθ angles slightly shift to larger values upon addition of cosolvents with the bigger drift found in the case of ternary solution water-urea-TMAO whose maximum *P*(cosθ) is around 0.318 (∼72°). This shows that for this system, there is a tilt angle deviation of about 18° from the dipole vector of water molecules, thus corresponding to a tangential orientation of one −OH arm to the solute surface.

Both urea and TMAO show larger cosθ values than water, except urea in *n*-hexane which exhibits downshifted negative cosθ values (−0.101, 96°) with intense and broad distributions. Moreover, TMAO presents consistent behavior with up-shifted cosθ values corresponding to a maximum average angle of about 65°. Thus, TMAO cosθ plots are centered around 65° implying that the vector formed by N–O atoms is on average tangential to the solute surface. This configuration enables to one methyl group for being solvated, while other are in contact with the bulk solution.

### 3.7 β2m Structural Properties

The conformational dynamics of β2m solvated in various cosolvent mixtures studied here can be assessed by monitoring the structural order parameters as shown in Figure 7 reporting the average root-mean-square-deviation (RMSD) from initial conformation Figure 7a, radius of gyration (*R*_g_) Figure 7b, and solvent accessible surface area (SASA) Figure 7c. The color code reads as follows: black (pure water H_2_O), red (water-urea), green (water-TMAO), and blue (water-urea-TMAO). It is rather convincing from Figure 7 that the addition of cosolvents urea and TMAO noticeably impairs the protein stability in relation to pure water, with a more prone effect induced by the addition of urea. It should, however, be stated that, under the conditions studied here and despite the conformational fluctuations seen, as evidenced by significant error drifts in RMSD plots (Figure 7a), no drastic secondary structural transition is reported (also see Section 3.9 below), as confirmed in part by the roughly equal trend in *R*_g_ plots (Figure 7b) with nearly equal average *R*_g_ values and relatively small standard deviations, likely witnessing the tight folded trait of the β2m structure. Overall, the stability of the studied protein in decrease order of stability in different cosolvents considered can be ranked as follows: water > water-TMAO > water-urea-TMAO > water-urea, highly consistent with the aggregation propensity pattern established for the hydrophobic solutes studied above (see Section 3.1). More intuitively, the dynamic overview of the studied system can be scrutinized by analyzing the time-dependent component of the previously depicted structural order parameters rather than the factual average terms, as reported in Figure SXIII.

**Figure 7:**
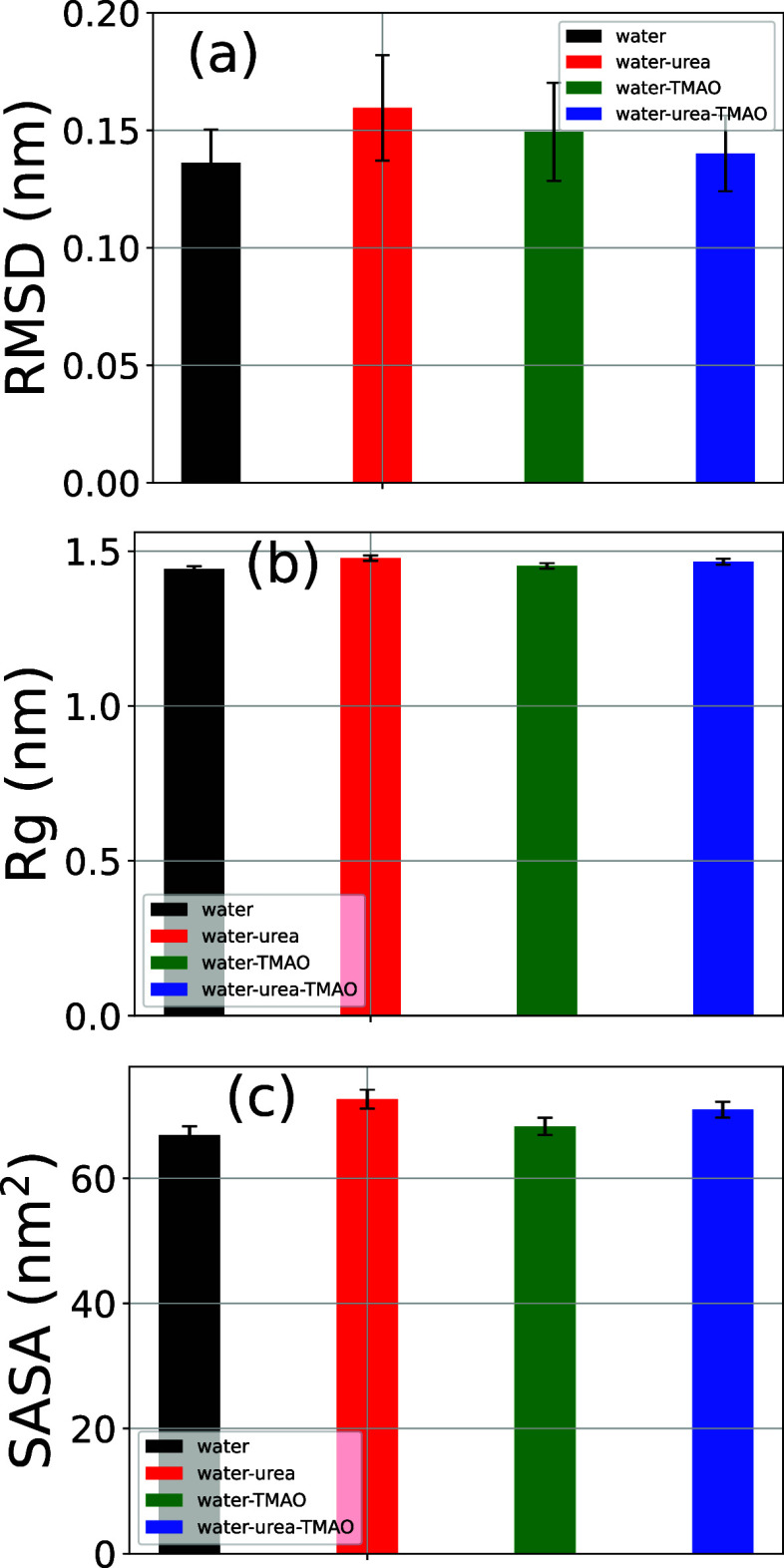
Average β2m root-mean-square-deviation (RMSD) from the initial conformation (a), radius of gyration (b), and solvent accessible surface area (SASA) (c) in different cosolvents studied in this work. Black bars depict the simulations in water, red ones those in 8.16 M water-urea, while green bars describe the simulations performed in 3.48 M water-TMAO, and blue color shows the data obtained in ternary mixture water-7.18 M urea-2.87 M TMAO. The error bars stand for standard deviations over the last 150 ns of simulation. The corresponding time-based plots are shown in Figure SXIII.

Figure SXIII displays the time-dependent structural changes of β2m in different solvent composition. In Figure SXIIIa, the change in the root-mean-square-deviation $\mathrm{RSMD}\left(t\right)=\sqrt{\sum {[r_{i}(t)-r_{i}(0)]}^{2}}$ from the initial conformation is displayed during the time evolution of the system where β2m is dispersed in water H_2_O (black curve), water-urea (red curve), water-TMAO (green curve), and water-urea-TMAO (blue curve). It is apparent that reasonable equilibration is achieved within tens of nanoseconds apart from the simulation in water-urea which stills exhibiting noticeable conformational rearrangements witnessing its enhanced propensity to denature β2m-folded state in particular and globular proteins in general.^2,11,70^ Moreover, the simulation in the ternary water-urea-TMAO solution results in fluctuating in phase with the pure water solution, evidencing the opposing cosolvent effects of TMAO and urea.^71^

Figure SXIIIb shows the radius of gyration $R_{g}=\sqrt{\underset{i}{\sum }m_{i}{[r_{i}-R_{CM}]}^{2}/M}$ (*m*_i_ is the mass of the *i*-th atom, ***R***_CM_ is the center of mass of the polymer chain, and *M* is the total mass), and Figure SXIIIc shows the solvent accessible surface area (SASA) using the algorithm devised by Eisenhaber et al.^72^ We note that SASA can be regarded as a proxy of the sum of the cavity and van der Waals contributions to the solvation free energy,^54^ and hence, its monitoring is particularly meaningful. The color code is the same as in Figure SXIIIa. It is worth recalling that as the protein conformation is tightly folded, its expanded volume is small, subsequently leading to relatively low *R*_g_ and SASA values. Figure SXIIIb,c agree well with the relative stability of β2m structure in the different solvent compositions, showing that the protein structure remains relatively more folded in pure water than in osmolyte solutions. In particular, a pronounced folded state is monitored in a pure water solution, the latter of which is slightly destabilized by the addition of TMAO, and further unfolded by urea. While consistent with the results shown in Figure SXIIIa, and with previous reported analysis on similar systems,^6,12,73^ this result further confirms the ability of TMAO to protect the folded state of β2m, thereby counteracting urea’s denaturation effect. As discussed below, our observation strongly agrees with the preferential binding mechanism of urea and the TMAO exclusion from the protein surface. This was even further assessed by computing the changes in the number of hydrogen bonds between β2m and the corresponding cosolvents herein studied as shown in Figure 10 as well as the corresponding number of cosolvents near the β2m surface as shown in Table 6.

### 3.8 Free Energy Landscape

The analysis reported in the previous section clearly shows how RMSD, radius of gyration *R*_*g*_, and SASA can all be used as possible ″reaction coordinates″ to track down and assess the folding/unfolding process. Accordingly, we can construct the relative energy landscape by monitoring their joint probability distribution and then the relative free energy landscape. Following a common choice in the literature, we selected *R*_*g*_ and SASA as reaction coordinates and computed the free energy landscape for all four solvents composition.

Figure 8 reports the results of this free energy landscape analysis, with the first row corresponding to water H_2_O (a) and water-urea (b) and the second row corresponding to water-TMAO (c) and water-urea-TMAO (d). It is noteworthy that in the case of pure water, a development of a well-localized minimum spatially located at low *R*_g_ and low SASA is observed. The same observation holds valid for system including water-TMAO binary solution, though with a slight shift outward to higher *R*_g_ and SASA values. Remarkably, conformations involving urea moieties are likely populating high *R*_g_ and SASA subregions, albeit with moderate shifts in *R*_g_-SASA space inasmuch as water-urea-TMAO is concerned. Thus, the protein structure exhibits some distortions upon addition of aqueous urea osmolyte witnessed by a less localized and sharp well depth, consistent to the view that urea induced protein denaturation, while the addition of TMAO reverts this action, thereby providing stabilizing effects. However, β2m is known to possess a highly stable structure; whether fluctuations induced by the action of urea and/or TMAO leads to some conformational structural changes is discussed below.

**Figure 8:**
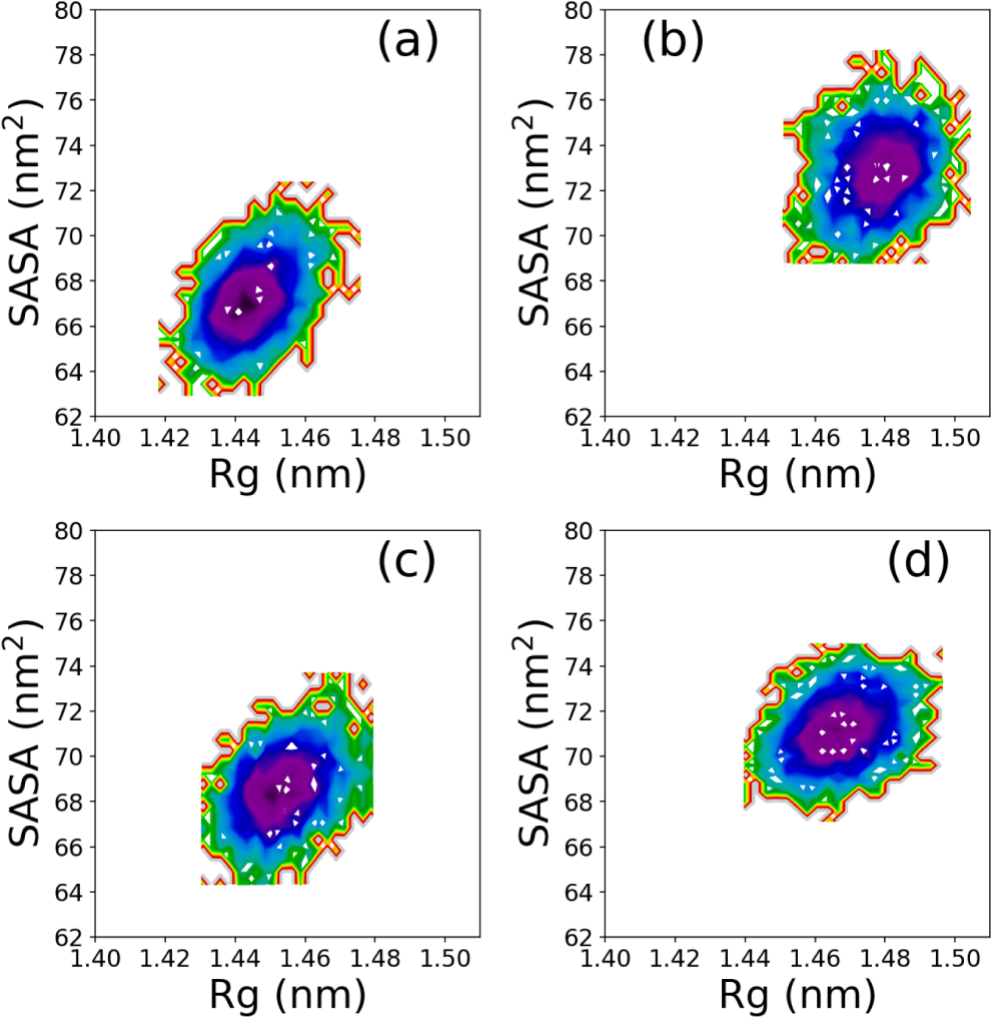
β2m free energy landscape (FEL) in water H_2_O and aqueous osmolyte mixtures using the radius of gyration *R*_g_ and SASA as reaction coordinates. From top to bottom, the FEL in water H_2_O (a), 8.16 M water-urea (b), 3.48 M water-TMAO (c), and ternary water-7.18 M urea-2.87 M TMAO solution (d) is reported. Note that the plots are on the same scale.

### 3.9 Secondary Structure Analysis

A comparison of conformational fluctuations for β2m in different solvent mixtures is reported in Figure SXII. While being fully consistent with the previous structural analyses in Figure SXIII, it is seen that urea drives the major conformational drift, significantly worth it in strands A and G and at the C terminal. Furthermore, noticeable fluctuations are depicted in the strands C, C’, and D (see nomenclature in Figure SXIV). The nearly same pattern emerges for the other solvents with the trend on solvent-induced stabilization of the native structure of β2m almost conserved. It should be recalled however that these marginally stable parts of β2m structure have already been pointed out to be important for general association properties and especially bounding to hydrophobic surfaces.^74^ Figure SXII principally emphasizes on local conformational fluctuations, but the extent to which this promotes global structural transition in β2m is analyzed below by computing the secondary structure change as a function of simulation time.

Figure SXIV reports the β2m secondary structural change as a function of the simulation time. From top to bottom, the secondary structure change in pure water, water-urea, water-TMAO, and water-urea-TMAO mixture is, respectively, displayed. Secondary structure analysis confirms and details the picture described above in Figure SXII, showing that the core of the protein involving strands B and F, including disulfide bridged residues Cys25···Cys80 and strands C and E, remains fairly stable. The most labile secondary structural element is strand D which is little preserved typically in water-urea-TMAO solution, whereas the terminal strands G display the ease to detach from the immunoglobulin fold.^28,74^ Taken together, the analysis of Figure SXIV does not indicate any significant secondary structure change consistent with the relative high stability of β2m fold at pH 7 ^33,34^ and the soft denaturation action of urea,^70^ at least for the concentration investigated herein (8.16 M) at the time scale achieved (200 ns).

### 3.10 β2m Preferential Interaction Coefficient, Γ

The analyses conducted so far on the hydrophobic hydrocarbon models including neopentane C_5_H_12_, cyclohexane cC_6_H_12_, and *n*-hexane *n*C_6_H_14_ have indicated the preference of amino −NH_2_ groups of urea to lie down parallel to the hydrophobic surface, whereas methyl −CH_3_ tails of TMAO side align concomitantly with the hydrophobic surface and toward the bulk phase mimicking an amphiphile object. Moreover, structural order parameters on a realistic hydrophobic solute, β2m, including RMSD, *R*_g_, and SASA, have clearly pointed out the enhanced propensity of urea to induce protein conformational unfolding, relative to pure water, while the addition of TMAO merely cancels out the denaturing action of urea, thereby restoring back the native state of the studied protein. Several mechanisms driving such conformational changes have been hypothesized including the preferential binding or exclusion,^12,73,75,76^ the TMAO depletion from the protein surface,^77^ and so on. To probe the interaction mode of β2m and the cosolutes understudied, we computed the preferential coefficient of urea and TMAO to β2m relative to pure water H_2_O as displayed in Figure 9.

**Figure 9:**
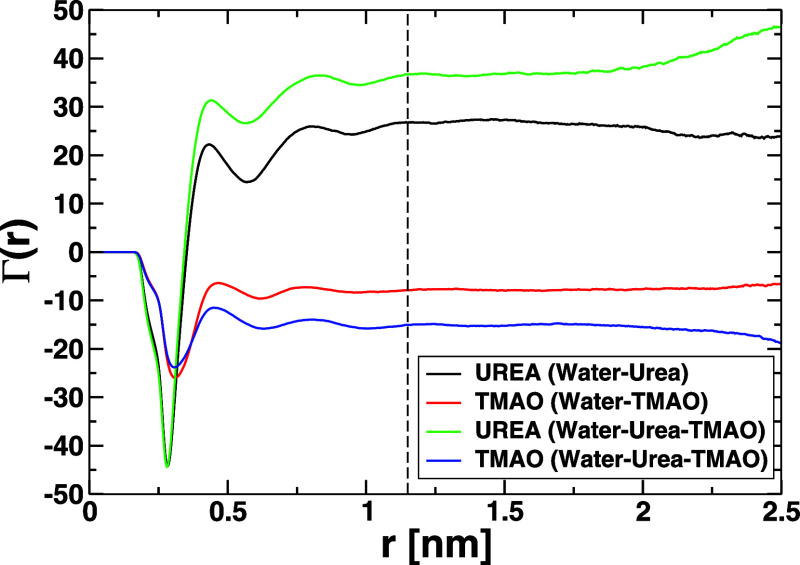
Preferential interaction coefficient (Γ) of urea and TMAO relative to pure water H_2_O with β2m surface at 300 K. Black lines correspond to urea in 8.16 M water-urea system, red lines correspond to TMAO in 3.48 M water-TMAO system, and green and blue lines correspond to urea and TMAO in ternary mixture water-7.18 M urea-2.87 M TMAO, respectively. The broken vertical line emphasizes the border between the local and the bulk domains of the solvated system and gives the value of the cutoff distance used to compute the value of Γ.

Figure 9 shows the distance-dependent preferential binding coefficient Γ of urea and TMAO to β2m relative to water. The black curve corresponds to the results of urea in water-urea, the red curve corresponds to those of TMAO in water-TMAO, and the green and blue curves show the results of urea and TMAO in the ternary mixture water-urea-TMAO, respectively. Urea and TMAO likely display firmly similar trends in both the binary and ternary phases analyzed here. More precisely, it is evidenced that urea moieties are preferentially excluded from the close vicinity of the protein surface in both water-urea and water-urea-TMAO within a distance range of about 0.35 nm before largely favorable protein-urea interactions turn on. Besides, a deep minimum in the preferential solvation plot of urea is found at ca. 0.28 nm, compatible with the first peak RDF in the Ow-Ow distribution (see Figure 5) pointing to the hydrogen-bonded first neighbor, thereby strongly suggesting a cluster of hydrogen-bonded water over urea’s accumulation at the inner solvation shell of β2m. It should be noted that, these data are qualitatively similar to the results of Khan and Nayeem^73^ on amyloidogenic heptapeptides and Khan and Nayeem^75^ on Aβ42 peptide (see Figure 8 in Khan and Nayeem^75^ ). Inasmuch as TMAO is concerned and as expected, its preferential exclusion from the protein surface is recorded with an enhanced exclusion pattern due to the addition of urea. This observation fits well, at least qualitatively, to the results of Canchi et al.^12^ relative to Trp-cage, ubiquitin, and lysozyme protein systems (see Figure 6 in Canchi et al.).^12^ Nonetheless, the distance-dependent Γ plot enables us to determine the appropriate value of the cutoff distance (see dashed line in Figure 9) useful to unequivocally estimate the preferential interaction coefficient Γ. This roughly corresponds to the point at which the plot flattens out. The computed values of Γ are reported in Table 5.

Taken together, our data strongly support the hypothesis of protein enhanced stability by TMAO as a result of its strong preferential exclusion nearby the protein surface (Γ < 0), whereas urea’s denaturation action is patterned to its strong and direct interaction with β2m surface (Γ > 0). This is further supported by the large number of urea moieties compared to TMAO and even water found in the first solvation of β2m as reported in Table 6. Furthermore, the direct interaction of urea and β2m is ultimately shown by deciphering the number of hydrogen bonds established between both entities along the simulation time as plotted in Figure 10.

**Figure 10:**
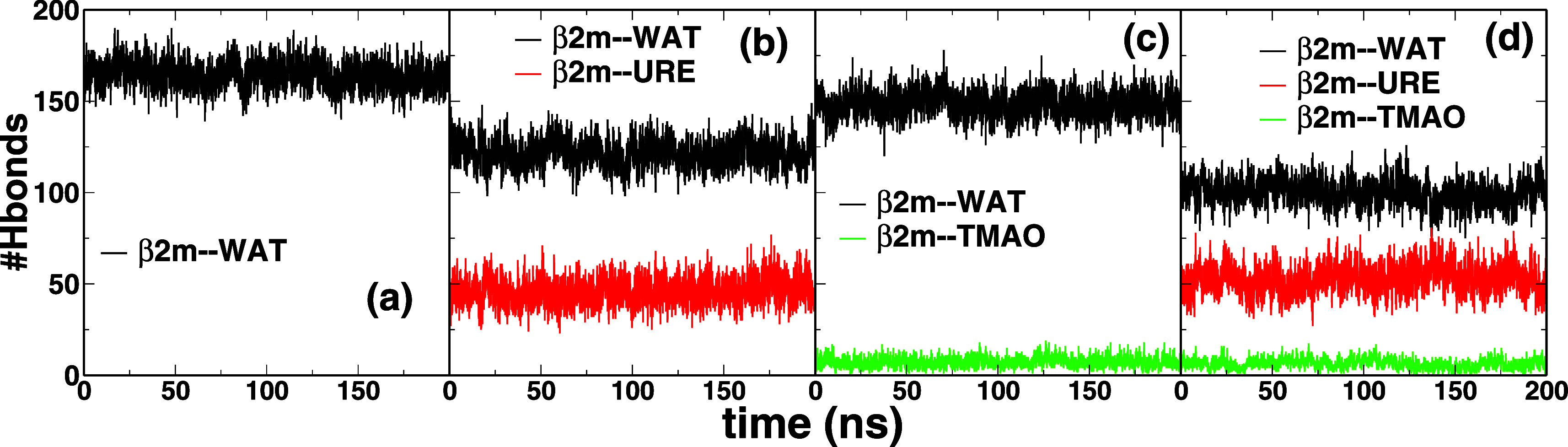
Time-based changes in the number of hydrogen bonds between β2m and the cosolvents in the different systems considered at 300 K. From left to right, the panels display the number of hydrogen bonds between β2m and cosolvents in pure water (a), in water-urea (b), in water-TMAO (c), and in ternary mixture water-urea-TMAO (d), respectively. Black lines correspond to the interactions between β2m and water, red to those between β2m and urea, and green to the β2m-TMAO counterpart.

Figure 10 reports the changes in the number of hydrogen bonds as a function of the simulation time. From left to right, the changes in the number of hydrogen bonds between β2m-water in pure water (a), β2m-water and β2m-urea in water-urea aqueous solution (b), β2m-water and β2m-TMAO in water-TMAO system (c), and β2m-water, β2m-urea, and β2m-TMAO in the ternary phase water-urea-TMAO (d). It clearly appears that the number of initial hydrogen bonds formed between β2m and water is shared among β2m-water and β2m-urea in the binary solution water-urea, owing to the direct interaction of urea with β2m, whereas the number of hydrogen bonds is nearly restored in the water-TMAO phase since only few hydrogen bonds are formed between TMAO and β2m, a signature of the exclusion of the former from the protein surface. This result is in perfect agreement with the analysis of the number of cosolvents found in the first solvation shell of β2m as reported in Table 6.

## 4 Conclusions

In this paper, we used molecular dynamics simulations at atomistic level and free energy calculations to provide insightful details on the effect of cosolvents urea and TMAO on the pairwise hydrophobic association scheme of three simple but representative hydrophobic models: one linear extended aliphatic alkane *n*-hexane *n*C_6_H_14_ ; one substituted aliphatic and commonly studied alkane neopentane C_5_H_12_; and one cyclic hydrocarbon cyclohexane cC_6_H_12_. These hydrophobic model systems were subsequently immersed in four different solvent models with varied composition: pure water, aqueous urea (water-8.16 M urea), aqueous TMAO (water-3.48 M TMAO), and mixed aqueous urea-TMAO ternary solution (water-7.18 M urea-2.87 M TMAO). Thereafter, we extent the knowledge to unravel the conformational properties and stability of a more realistic hydrophobic solute, β2-microglobulin, a paradigmatic protein model for amyloid studies.^30^ Among the hydrocarbons studied here, *n*-hexane *n*C_6_H_14_ is the only one showing a peculiar hydrophobic interaction scheme likely pertaining to its extended structure and less spherical shape that constrains the approach of two molecules in a more specific orientation compared to neopentane C_5_H_12_ and cyclohexane cC_6_H_12_. We show that the contact minimum configurations are largely stabilized by entropy at the opposite of solvent-separated minimum configurations, which are dominantly enthalpically driven, induced by water hydrogen bonding. Besides, while our data firmly support the dehydration of the hydrophobic solutes owing to their preferential binding with osmolytes urea and TMAO (Γ > 0), it is further found that the PMFs at the contact minimum configurations are deeper (more negative) in pure water than in osmolyte mixture phases, showing that the hydrophobic clusters do not completely disentangle in aqueous urea and TMAO solutions, but instead, these latter act as a *gum* bridging between pairwise hydrophobic moieties holding them together, well in accord with the results of Lee and van der Vegt.^19^ In general, the picture that emerges from the simulations of β2m in osmolytes urea and TMAO is that, albeit the protein undergoes noticeable conformational fluctuations, no drastic structural transition is recorded, also taking into account the relatively high stability of β2m fold under standard conditions.^33,34^ Moreover, TMAO displays the most coherent feature, mainly acting against urea chemical denaturation effects. Our results indicate that TMAO protects β2m-folded state by its strong preferential exclusion from the close vicinity of the β2m surface (Γ_TMAO_ < 0), whereas as in many protein models, urea denaturation action conveys a more complex pattern, being initially excluded from the close inner solvation shell of β2m before systematically accumulating around the protein beyond a threshold distance of ∼ 0.35 nm. Furthermore, our results disclose a large number of urea moieties around the first solvation shell of β2m, even making favorable hydrogen bonds with the latter. Hence, our results are compatible with both direct and indirect β2m urea-induced denaturation models, since it is found to be excluded from the close surrounding of the protein (Γ_UREA_ < 0) and to accumulate systematically around it from about 0.35 nm (Γ_UREA_ > 0).

## Supplementary Material



## Notes

Additional results, including figures and tables (PDF)

## References

[1] Anfinsen C. B. (1972). The formation
and stabilization of protein structure. Biochem.
J..

[2] Gazi R., Maity S., Jana M. (2023). Conformational
Features and Hydration
Dynamics of Proteins in Cosolvents: A Perspective from Computational
Approaches. ACS Omega.

[3] Timasheff S. N. (1993). The Control
of Protein Stability and Association by Weak Interactions with Water:
How Do Solvents Affect These Processes?. Annu.
Rev. Biophys..

[4] Giri, S. ; Singh, P. ; Biswas, M. ; Mishra, R. ; Poddar, N. K. Cellular Osmolytes: from Chaperoning Protein Folding to Clinical Perspectives; Singh, L. R. ; Dar, T. A. ; Kumari, K. , Eds.; Springer Nature Singapore: Singapore, 2024; pp. 129–160.

[5] Ganguly P., van der Vegt N. F. A., Shea J.-E. (2016). Hydrophobic Association in Mixed
Urea-TMAO Solutions. J. Phys. Chem. Lett..

[6] Ganguly P., Polak J., van der Vegt N. F. A., Heyda J., Shea J.-E. (2020). Protein
Stability in TMAO and Mixed Urea-TMAO Solutions. J. Phys. Chem. B.

[7] Sarma R., Paul S. (2011). Hydrophobic interactions
in presence of osmolytes urea and trimethylamine-N-oxide. J. Chem. Phys..

[8] Sarma R., Paul S. (2012). Association of Small
Hydrophobic Solute in Presence of the Osmolytes
Urea and Trimethylamine-N-oxide. J. Phys. Chem.
B.

[9] Paul S., Patey G. N. (2008). Hydrophobic Interactions in Urea-Trimethylamine-N-oxide
Solutions. J. Phys. Chem. B.

[10] Baynes B. M., Trout B. L. (2003). Proteins in Mixed Solvents: A Molecular-Level Perspective. J. Phys. Chem. B.

[11] Canchi D. R., Paschek D., García A. E. (2010). Equilibrium Study of Protein Denaturation
by Urea. J. Am. Chem. Soc..

[12] Canchi D. R., Jayasimha P., Rau D. C., Makhatadze G. I., Garcia A. E. (2012). Molecular Mechanism for the Preferential Exclusion
of TMAO from Protein Surfaces. J. Phys. Chem.
B.

[13] Rodríguez-Ropero F., Rötzscher P., van der Vegt N. F. A. (2016). Comparison of Different TMAO Force
Fields and Their Impact on the Folding Equilibrium of a Hydrophobic
Polymer. J. Phys. Chem. B.

[14] Canchi D. R., García A. E. (2013). Cosolvent Effects on Protein Stability. Annu. Rev. Phys. Chem..

[15] Rani A., Jayaraj A., Jayaram B., Pannuru V. (2016). Trimethylamine-N-oxide
switches from stabilizing nature: A mechanistic outlook through experimental
techniques and molecular dynamics simulation. Sci. Rep..

[16] Bankir, L. Urea Transporters; Yang, B. ; Sands, J. M. , Eds.; Springer: Netherlands, 2014; pp. 193–226.

[17] Tanford C. (1997). How protein
chemists learned about the hydrophobic factor. Protein Sci.

[18] Daggett V. (2006). Protein Folding-Simulation. Chem. Rev..

[19] Lee M.-E., van der Vegt N. F. A. (2006). Does
Urea Denature Hydrophobic Interactions?. J.
Am. Chem. Soc..

[20] Dongmo
Foumthuim C. J., Carrer M., Houvet M., Škrbić T., Graziano G., Giacometti A. (2020). Can the roles of polar and non-polar
moieties be reversed in non-polar solvents?. Phys. Chem. Chem. Phys..

[21] Dongmo
Foumthuim C. J., Arcangeli T., Škrbić T., Giacometti A. (2024). Solvent quality and nonbiological oligomer folding:
revisiting conventional paradigms. Soft Matter.

[22] Dongmo
Foumthuim C. J., Giacometti A. (2023). Solvent quality and solvent polarity
in polypeptides. Phys. Chem. Chem. Phys..

[23] Floege J., Bartsch A., Schulze M., Shaldon S., Koch K. M., Smeby L. C. (1991). Clearance and synthesis
rates of *β*2-microglobulin in patients undergoing
hemodialysis and in normal
subjects. J. Lab. Clin. Med..

[24] Scarpioni R., Ricardi M., Albertazzi V., De Amicis S., Rastelli F., Zerbini L. (2016). Dialysis-related amyloidosis:
challenges
and solutions. Int. J. Nephrol. Renovasc. Dis..

[25] Gejyo F., Yamada T., Odani S., Nakagawa Y., Arakawa M., Kunitomo T., Kataoka H., Suzuki M., Hirasawa Y., Shirahama T. (1985). A new form of amyloid protein associated with
chronic hemodialysis was identified as *β*2-microglobulin. Biochem. Biophys. Res. Commun..

[26] Yamamoto S., Gejyo F. (2005). Historical background
and clinical treatment of dialysis-related
amyloidosis. Biochim. Biophys. Acta, Proteins
Proteomics.

[27] Naiki H., Okoshi T., Ozawa D., Yamaguchi I., Hasegawa K. (2016). Molecular pathogenesis of human amyloidosis:
Lessons
from *β*2-microglobulin-related amyloidosis. Pathol. Int..

[28] Fogolari F., Corazza A., Varini N., Rotter M., Gumral D., Codutti L., Rennella E., Viglino P., Bellotti V., Esposito G. (2011). Molecular dynamics simulation of *β*2-microglobulin in denaturing and stabilizing conditions. Proteins: struct., Funct., Bioinf..

[29] Becker J. W., Reeke G. N. (1985). Three-dimensional
structure of *β*2-microglobulin. Proc. Int. Acad. Sci..

[30] Saper M., Bjorkman P., Wiley D. (1991). Refined structure of the human histocompatibility
antigen HLA-A2 at 2.6 Å resolution. J.
Mol. Biol..

[31] Verdone G., Corazza A., Viglino P., Pettirossi F., Giorgetti S., Mangione P., Andreola A., Stoppini M., Bellotti V., Esposito G. (2002). The solution structure
of human *β*2-microglobulin reveals the prodromes
of its amyloid
transition. Protein Sci..

[32] Ohta Y., Shiina T., Lohr R. L., Hosomichi K., Pollin T. I., Heist E. J., Suzuki S., Inoko H., Flajnik M. F. (2011). Primordial Linkage of *β*2-Microglobulin
to the MHC. J. Immunol..

[33] Bellotti V., Stoppini M., Mangione P., Sunde M., Robinson C., Asti L., Brancaccio D., Ferri G. (1998). *β*2-microglobulin can be refolded into a native
state from ex vivo
amyloid fibrils. Eur. J. Biochem..

[34] Myers S. L., Jones S., Jahn T. R., Morten I. J., Tennent G. A., Hewitt E. W., Radford S. E. (2006). A Systematic
Study of the Effect
of Physiological Factors on *β*2-Microglobulin
Amyloid Formation at Neutral pH. Biochemistry.

[35] Dilip H. N., Chakraborty D. (2020). Effect of
cosolvents in the preferential binding affinity
of water in aqueous solutions of amino acids and amides. J. Mol. Liq..

[36] Su Z., Dias C. L. (2019). Individual and combined
effects of urea and trimethylamine
N-oxide (TMAO) on protein structures. J. Mol.
Liq..

[37] Jorgensen W. L., Chandrasekhar J., Madura J. D., Impey R. W., Klein M. L. (1983). Comparison
of simple potential functions for simulating liquid water. J. Chem. Phys..

[38] Weerasinghe S., Smith P. E. (2003). A Kirkwood-Buff Derived Force Field for Mixtures of
Urea and Water. J. Phys. Chem. B.

[39] Kast K. M., Brickmann J., Kast S. M., Berry R. S. (2003). Binary Phases of
Aliphatic N-Oxides and Water: Force Field Development and Molecular
Dynamics Simulation. J. Phys. Chem. A.

[40] Hölzl C., Kibies P., Imoto S., Noetzel J., Knierbein M., Salmen P., Paulus M., Nase J., Held C., Sadowski G. (2019). Structure
and thermodynamics of aqueous urea
solutions from ambient to kilobar pressures: From thermodynamic modeling,
experiments, and first principles simulations to an accurate force
field description. Biophys. Chem..

[41] Martin M. G., Siepmann J. I. (1999). Novel Configurational-Bias
Monte Carlo Method for Branched
Molecules. Transferable Potentials for Phase Equilibria. 2. United-Atom
Description of Branched Alkanes. J. Phys. Chem.
B.

[42] Jorgensen W. L., Madura J. D., Swenson C. J. (1984). Optimized intermolecular potential
functions for liquid hydrocarbons. J. Am. Chem.
Soc..

[43] Plimpton S. (1995). Fast Parallel
Algorithms for Short-Range Molecular Dynamics. J. Comput. Biophys. Chem..

[44] van
der Vegt N. F. A., Lee M.-E., Trzesniak D., van Gunsteren W. F. (2006). Enthalpy-Entropy Compensation in the Effects of Urea
on Hydrophobic Interactions. J. Phys. Chem.
B.

[45] Smith D. E., Zhang L., Haymet A. D. J. (1992). Entropy of association
of methane
in water: a new molecular dynamics computer simulation. J. Am. Chem. Soc..

[46] Smith D. E., Haymet A. D. J. (1993). Free energy, entropy, and internal energy of hydrophobic
interactions: Computer simulations. J. Chem.
Phys..

[47] Ikeguchi M., Nakamura S., Shimizu K. (2001). Molecular
Dynamics Study on Hydrophobic
Effects in Aqueous Urea Solutions. J. Am. Chem.
Soc..

[48] Su Z., Ravindhran G., Dias C. L. (2018). Effects of Trimethylamine-N-oxide
(TMAO) on Hydrophobic and Charged Interactions. J. Phys. Chem. B.

[49] Southall N.
T., Dill K. A. (2002). Potential
of mean force between two hydrophobic solutes
in water. Biophys. Chem..

[50] Shimizu S., Chan H. S. (2000). Temperature dependence of hydrophobic
interactions:
A mean force perspective, effects of water density, and nonadditivity
of thermodynamic signatures. J. Chem. Phys..

[51] Czaplewski C., Rodziewicz-Motowidło S., Dabal M., Liwo A., Ripoll D. R., Scheraga H. A. (2003). Molecular
simulation study of cooperativity
in hydrophobic association: clusters of four hydrophobic particles. Biophys. Chem..

[52] Raschke T. M., Tsai J., Levitt M. (2001). Quantification of the hydrophobic
interaction by simulations of the aggregation of small hydrophobic
solutes in water. Proc. Int. Acad. Sci..

[53] Oostenbrink C., van Gunsteren W. F. (2005). Methane
clustering in explicit water: effect of urea
on hydrophobic interactions. Phys. Chem. Chem.
Phys..

[54] Leach, A. R. Molecular modelling: principles and applications; Pearson education, 2001.

[55] Schmid N., Eichenberger A. P., Choutko A., Riniker S., Winger M., Mark A. E., van Gunsteren W. F. (2011). Definition and testing of the GROMOS
force-field versions 54A7 and 54B7. Eur. Biophys.
J..

[56] Berendsen H. J. C., Grigera J. R., Straatsma T. P. (1987). The missing
term in effective pair
potentials. J. Phys. Chem..

[57] Lindorff-Larsen K., Piana S., Palmo K., Maragakis P., Klepeis J. L., Dror R. O., Shaw D. E. (2010). Improved
side-chain
torsion potentials for the Amber ff99SB protein force field. Proteins: struct., Funct., Bioinf..

[58] Bussi G., Donadio D., Parrinello M. (2007). Canonical
sampling through velocity
rescaling. J. Chem. Phys..

[59] Hess B., Bekker H., Berendsen H. J. C., Fraaije J. G. E. M. (1997). LINCS: A linear
constraint solver for molecular simulations. J. Comput. Chem..

[60] Parrinello M., Rahman A. (1981). Polymorphic transitions
in single crystals: A new molecular
dynamics method. J. Appl. Phys..

[61] Lemkul J. A. (2024). Introductory
Tutorials for Simulating Protein Dynamics with GROMACS. J. Phys. Chem. B.

[62] Ben-Naim, A. Y. Hydrophobic interactions; Springer Science & Business Media, 2012.

[63] Ghosh T., García A. E., Garde S. (2001). Molecular Dynamics Simulations of
Pressure Effects on Hydrophobic Interactions. J. Am. Chem. Soc..

[64] Zangi R., Berne B. J. (2006). Aggregation and
Dispersion of Small Hydrophobic Particles
in Aqueous Electrolyte Solutions. J. Phys. Chem.
B.

[65] Athawale M. V., Goel G., Ghosh T., Truskett T. M., Garde S. (2007). Effects of
lengthscales and attractions on the collapse of hydrophobic polymers
in water. Proc. Int. Acad. Sci..

[66] Dias C. L., Ala-Nissila T., Wong-Ekkabut J., Vattulainen I., Grant M., Karttunen M. (2010). The hydrophobic
effect and its role
in cold denaturation. Cryobiology.

[67] Hajari T., Dixit M., Yadav H. O. S. (2022). Hydrophobic association
and solvation
of neopentane in urea, TMAO and urea-TMAO solutions. Phys. Chem. Chem. Phys..

[68] Dixit M., Hajari T., Tembe B. (2016). The effect of urea and taurine osmolytes
on hydrophobic association and solvation of methane and neopentane
molecules. J. Mol. Liq..

[69] Athawale M. V., Dordick J. S., Garde S. (2005). Osmolyte Trimethylamine-N-Oxide
Does
Not Affect the Strength of Hydrophobic Interactions: Origin of Osmolyte
Compatibility. Biophys. J..

[70] Paladino A., Vitagliano L., Graziano G. (2023). The Action of Chemical Denaturants:
From Globular to Intrinsically Disordered Proteins. Biology.

[71] Graziano G. (2011). How does trimethylamine
N-oxide counteract the denaturing activity of urea?. Phys. Chem. Chem. Phys..

[72] Eisenhaber F., Lijnzaad P., Argos P., Sander C., Scharf M. (1995). The double
cubic lattice method: Efficient approaches to numerical integration
of surface area and volume and to dot surface contouring of molecular
assemblies. J. Comput. Chem..

[73] Khan A., Nayeem S. M. (2020). Effect of TMAO and
Urea on Dimers and Tetramers of
Amyloidogenic Heptapeptides (23FGAILSS29). ACS
Omega.

[74] Dongmo
Foumthuim C. J., Corazza A., Esposito G., Fogolari F. (2017). Molecular
dynamics simulations of *β*2-microglobulin interaction
with hydrophobic surfaces. Mol. BioSyst..

[75] Khan A., Nayeem S. M. (2023). Stability of the
A*β*42 Peptide
in Mixed Solutions of Denaturants and Proline. J. Phys. Chem. B.

[76] Meersman F., Bowron D., Soper A. K., Koch M. H. (2009). Counteraction of
Urea by Trimethylamine N-Oxide Is Due to Direct Interaction. Biophys. J..

[77] Folberth A., van der Vegt N. F. A. (2022). Influence of TMAO and Pressure on
the Folding Equilibrium
of TrpCage. J. Phys. Chem. B.

