# Supplementary material for: Hydrophobic Interactions in Aqueous Osmolyte Solutions: Thermodynamics of Solvation and Implication on Protein Stability
Source: J Phys Chem B. 2025 May 19;129(21):5150–65. doi: 10.1021/acs.jpcb.5c00785 (PMC12128031; doi:10.1021/acs.jpcb.5c00785)
Supplement: Supplementary file 1 [file jp5c00785_si_001.pdf]

Hydrophobic Interactions in Aqueous Osmolyte Solutions : Thermodynamics of Solvation and Implication on Protein Stability Supplementary Information

Cedrix J. Dongmo Foumthuim*

Department of Chemical Sciences (DisC), University of Padova, via Marzolo 1, 35131 Padova (Italy)

E-mail: cedrix85@gmail.com,cedrix.dongmo@unipd.it

Contents

Table SI - Average values of temperature and pressure for each system studied here. For the hydrophobic solutes including neopentane, cyclohexane and n-hexane, the simulations were performed at constant temperature NVT, thus monitoring the pressure is not relevant. S6

Figure SI - Convergence plots of temperature for each hydrophobic solute studied here in each of the multiphase system analyzed. From left to right the results of neopentane C_5H_{12} (a)$\cdots$(d), cyclohexane cC_6H_{12} (e)$\cdots$(h) and n-hexane nC_6H_{14} (i)$\cdots$(l) are respectively displayed. From top to bottom results in different solvent composition namely pure water, 8.16M water-urea, 3.48M water-TMAO, and water/7.18M urea/2.87M TMAO are respectively displayed. Gray lines show the fluctuations over the temperature. The running average plots are shown in black corresponding to 273.15 K, while red lines point to 298.15 K, and green ones refer to 323.15 K. The corresponding average values are summarized in Table SI below. S7

Figure SII - Convergence plots of temperature (a)$\cdots$(d) (top) and pressure (e)$\cdots$(h) (bottom) of β2m in each of the solvent mixtures studied. From left to right the results obtained in water, 8.16M water-urea, 3.48M water-TMAO, and water/7.18M urea/2.87M TMAO are respectively displayed. Gray lines show the fluctuations over the order parameter considered while red lines are their corresponding running averages. The corresponding average values are summarized in Table SI below. S8

Figure SIII - Temperature dependence of the solvation free energy ΔG_{solv} from gas to water H_2O in each of the hydrocarbons studied in this work neopentane C_5H_{12} (a), cyclohexane cC_6H_{12} (b) and n-hexane nC_6H_{14} (c). S8

Figure SIV - Temperature dependence of the potentials of mean force of neopentane C_5H_{12} (a)$\cdots$(d) (left), cyclohexane cC_6H_{12} (e)$\cdots$(h) (middle) and of n-hexane nC_6H_{14} (i)$\cdots$(l) (right) molecules. From top to bottom panels show the results in water solution, 8.16M water-urea, 3.48M water-TMAO, and water/7.18M urea/2.87M TMAO ternary mixture, respectively. The black line refers to the temperature of 273.15K, the red to 298.15K and the blue one to 323.15K. S9

Figure SV - Preferential interaction (Γ) of urea and TMAO with each hydrophobic hydrocarbon investigated here at 298.15 K. Neopentane C_5H_{12} results are shown in (a), cyclohexane cC_6H_{12} one in (b) and of n-hexane nC_6H_{14} counterpart in (c). Black lines correspond to urea in 8.16M water-urea system, red lines to TMAO in 3.48M water-TMAO system, green and blue lines to urea and TMAO in ternary mixture water/7.18M urea/2.87M TMAO, respectively. The vertical intercepted line marks the border between the local and the bulk domains and correspond to the cutoff value used to determine Γ. A negative value of the total preferential binding coefficient Γ implies the depletion of the considered cosolute. S10

Figure SVI - Solute-solute Potential of Mean Force (ΔG) at 298.15 K for each of the hydrocarbons studied here in different solvent phases. Standard deviations are reported as vertical error bars. From left to right the results of neopentane C_5H_{12} (a)$\cdots$(d), cyclohexane cC_6H_{12} (e)$\cdots$(h) and n-hexane nC_6H_{14} (i)$\cdots$(l) are respectively displayed. From top to bottom results in different solvent mixtures namely pure water (a, e, i), 8.16M water-urea (b, f, j), 3.48M water-TMAO (c, g, k), and water/7.18M urea/2.87M TMAO (d, g, k) are respectively displayed. S11

Figure SVII - Entropic contribution (TΔS) to the solute-solute Potential of Mean Force at 298.15 K for each of the hydrocarbons studied here in different solvent phases. Standard deviations are reported as vertical error bars. From left to right the results of neopentane C_5H_{12} (a)$\cdots$(d), cyclohexane cC_6H_{12} (e)$\cdots$(h) and *n*-hexane nC_6H_{14} (i)$\cdots$(l) are respectively displayed. From top to bottom results in different solvent mixtures namely pure water (a, e, i), 8.16M water-urea (b, f, j), 3.48M water-TMAO (c, g, k), and water/7.18M urea/2.87M TMAO (d, g, k) are respectively displayed. S12

Figure SVIII - Enthalpic contribution (ΔH) to the solute-solute Potential of Mean Force at 298.15 K for each of the hydrocarbons studied here in different solvent phases. Standard deviations are reported as vertical error bars. From left to right the results of neopentane C_5H_{12} (a)$\cdots$(d), cyclohexane cC_6H_{12} (e)$\cdots$(h) and *n*-hexane nC_6H_{14} (i)$\cdots$(l) are respectively displayed. From top to bottom results in different solvent mixtures namely pure water (a, e, i), 8.16M water-urea (b, f, j), 3.48M water-TMAO (c, g, k), and water/7.18M urea/2.87M TMAO (d, g, k) are respectively displayed. S13

Figure SIX - Schematic illustration of the definition of the angle θ used to compute the angular distribution of co-solutes in the solvation shell in the case of neopentane. In (a) the angle formed between solute and water, in (b) between solute and urea and in (c) between solute and TMAO. S14

Figure SX - Angular distributions of co-solvents around the hydrophobic solutes in the inner solvation shell. The latter is taken here as the distance within 0.5 nm from the solute atoms. Co-solvents orientation around neopentane C_5H_{12} are shown on the left (a)$\cdots$(d), around cyclohexane cC_6H_{12} in the middle (e)$\cdots$(h), and around *n*-hexane nC_6H_{14} on the right (i)$\cdots$(l). From top to bottom panels show the results in water solution, 8.16M water-urea, 3.48M water-TMAO, and water/7.18M urea/2.87M TMAO ternary mixture, respectively. The black line refers to the angular distribution of water, red line to that of urea and green line to TMAO. S15

Figure SXI - Schematic view of the topology of β2m alongside with the secondary structure description. S16
Figure SXII - Residue average root mean square fluctuations of backbone atoms. Black line depicts the simulation water, red line the one in 8.16M water-urea, while green line describes the simulation performed in 3.48M water-TMAO, and blue line shows the data obtained in ternary mixture water/7.18M urea/2.87M TMAO. S16
Figure SXIII - Conformational structural dynamics of β2m in different systems studied in this work. From top to bottom the time-dependent changes of the RMSD, Rg and SASA are displayed, respectively. Black line depicts the simulations in water, red line those in 8.16M water-urea, while green line describes the simulations performed in 3.48M water-TMAO, and blue line shows the data obtained in ternary mixture water/7.18M urea/2.87M TMAO. S17
Figure SXIV - β2m secondary structure change as a function of simulation time. From top to bottom the secondary structure change of β2m in water H_2O (a), 8.16M water/urea (b), 3.48M water/TMAO (c) and ternary mixture water/7.18M urea/2.87M TMAO (d) solution are reported, respectively. Color coding for different secondary structural elements are shown at the bottom panel. . . S18

Table SI: Average values of temperature and pressure for each system studied here. For the hydrophobic solutes including neopentane, cyclohexane and n-hexane, the simulations were performed at constant temperature NVT, thus monitoring the pressure is not relevant.

Systems	Cosolvents	T (K)	P (bar)
Neopentane	Water	273.143 ± 2.48619	−//−
		298.147 ± 2.71464	−//−
		323.149 ± 2.94425	−//−
	Water-Urea	273.147 ± 2.04704	−//−
		298.147 ± 2.23462	−//−
		323.145 ± 2.42142	−//−
	Water-TMAO	273.149 ± 2.12811	−//−
		298.150 ± 2.32333	−//−
		323.147 ± 2.51754	−//−
	Water-Urea-TMAO	273.149 ± 1.83534	−//−
		298.149 ± 2.00318	−//−
		323.150 ± 2.17008	−//−
Cyclohexane	Water	273.149 ± 2.48591	−//−
		298.151 ± 2.71284	−//−
		323.155 ± 2.9396	−//−
	Water-Urea	273.150 ± 2.0484	−//−
		298.146 ± 2.23577	−//−
		323.147 ± 2.42451	−//−
	Water-TMAO	273.147 ± 2.12654	−//−
		298.150 ± 2.32096	−//−
		323.154 ± 2.51503	−//−
	Water-Urea-TMAO	273.151 ± 1.83354	−//−
		298.150 ± 2.00081	−//−
		323.151 ± 2.16969	−//−
$n-$hexane	Water	273.15 ± 2.48558	−//−
		298.149 ± 2.71196	−//−
		323.151 ± 2.94135	−//−
	Water-Urea	273.147 ± 2.0479	−//−
		298.151 ± 2.23571	−//−
		323.145 ± 2.42072	−//−
	Water-TMAO	273.149 ± 2.12661	−//−
		298.148 ± 2.32285	−//−
		323.152 ± 2.51681	−//−
	Water-Urea-TMAO	273.148 ± 1.83342	−//−
		298.148 ± 2.00233	−//−
		323.150 ± 2.16853	−//−
β2m	Water	299.948 ± 1.32604	1.06348 ± 105.592
	Water-Urea	300.018 ± 1.29932	0.738108 ± 177.877
	Water-TMAO	300.281 ± 1.44579	1.3171 ± 275.311
	Water-Urea-TMAO	300.033 ± 1.26059	0.985484 ± 189.719

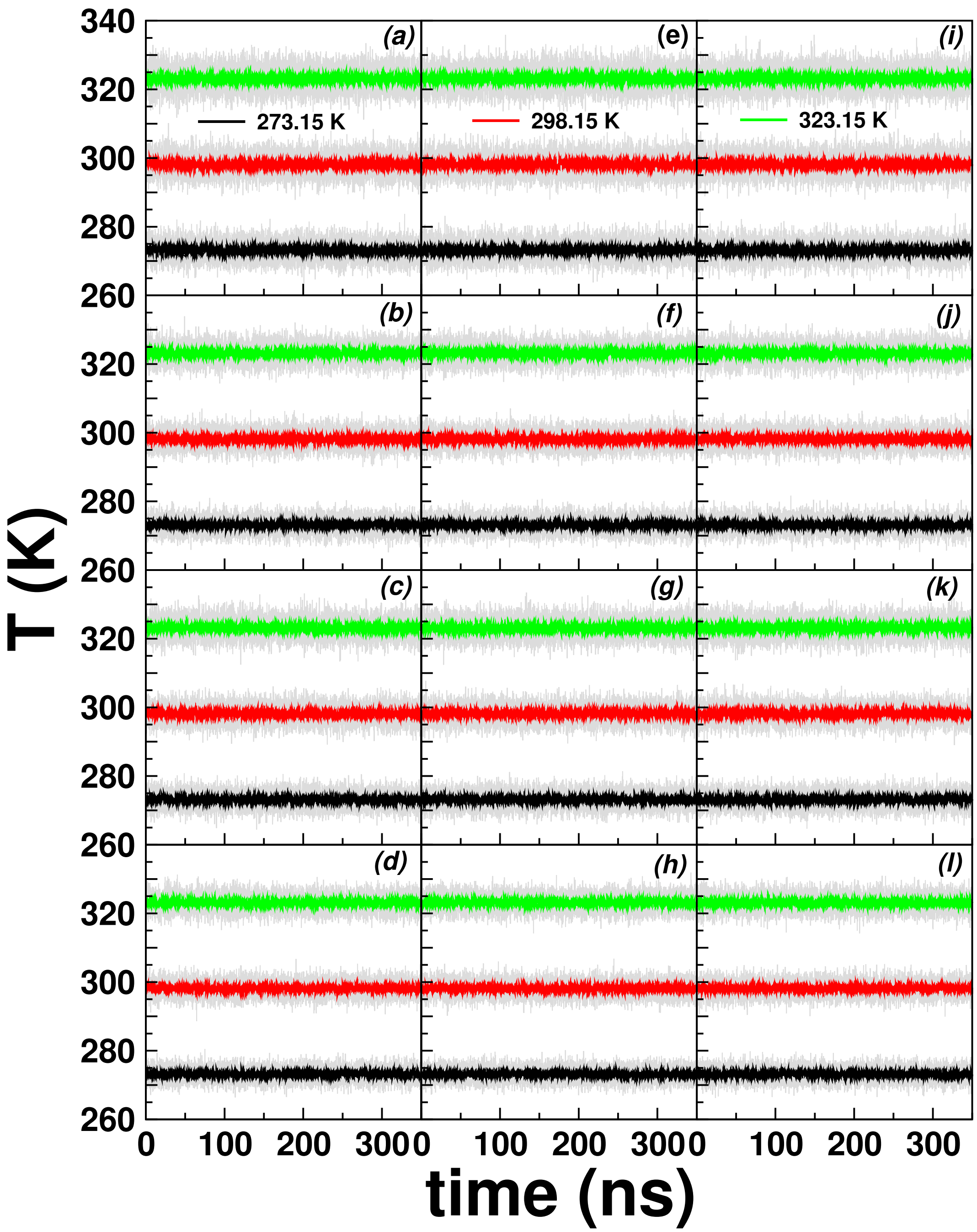

Figure SI: Convergence plots of temperature for each hydrophobic solute studied here in each of the multiphase system analyzed. From left to right the results of neopentane C_5H_{12} (a)$\cdots$(d), cyclohexane cC_6H_{12} (e)$\cdots$(h) and n-hexane nC_6H_{14} (i)$\cdots$(l) are respectively displayed. From top to bottom results in different solvent composition namely pure water, 8.16M water-urea, 3.48M water-TMAO, and water/7.18M urea/2.87M TMAO are respectively displayed. Gray lines show the fluctuations over the temperature. The running average plots are shown in black corresponding to 273.15 K, while red lines point to 298.15 K, and green ones refer to 323.15 K. The corresponding average values are summarized in Table SI below.

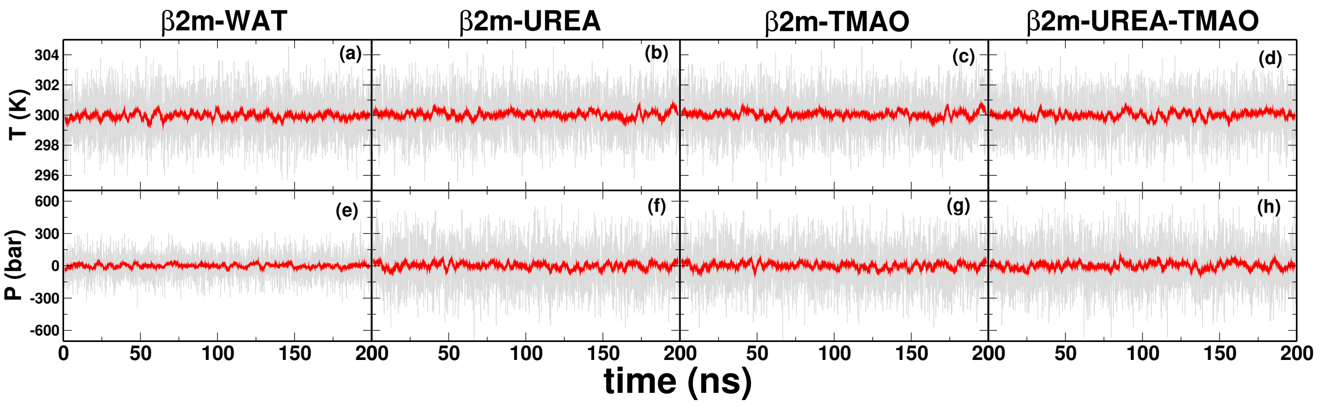

Figure SII: Convergence plots of temperature (a)$\cdots$(d) (top) and pressure (e)$\cdots$(h) (bottom) of β2m in each of the solvent mixtures studied. From left to right the results obtained in water, 8.16M water-urea, 3.48M water-TMAO, and water/7.18M urea/2.87M TMAO are respectively displayed. Gray lines show the fluctuations over the order parameter considered while red lines are their corresponding running averages. The corresponding average values are summarized in Table SI below.

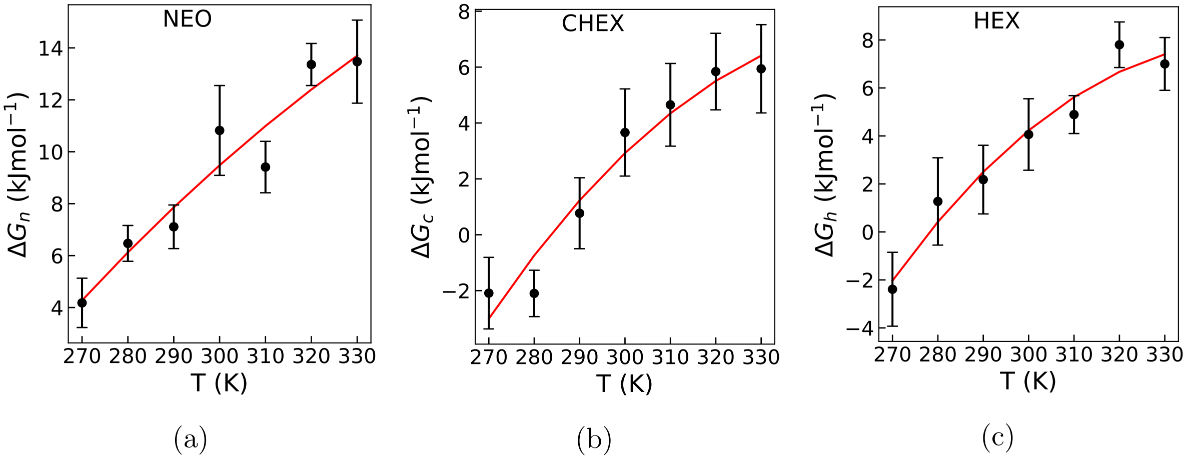

Figure SIII: Temperature dependence of the solvation free energy ΔG_{solv} from gas to water H_2O in each of the hydrocarbons studied in this work neopentane C_5H_{12} (a), cyclohexane cC_6H_{12} (b) and n-hexane nC_6H_{14} (c).

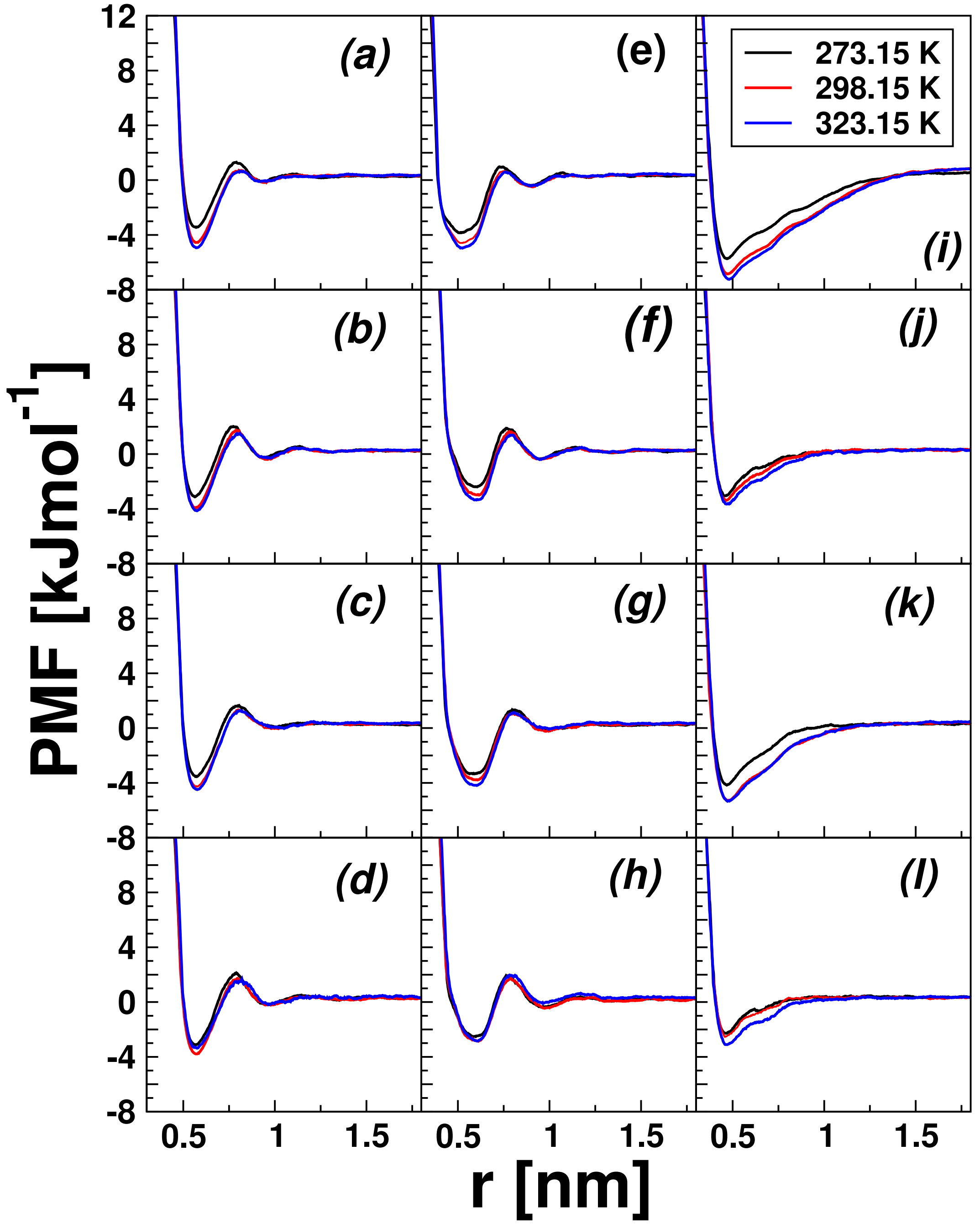

Figure SIV: Temperature dependence of the potentials of mean force of neopentane C_5H_{12} (a)$\cdots$(d) (left), cyclohexane cC_6H_{12} (e)$\cdots$(h) (middle) and of n-hexane nC_6H_{14} (i)$\cdots$(l) (right) molecules. From top to bottom panels show the results in water solution, 8.16M water-urea, 3.48M water-TMAO, and water/7.18M urea/2.87M TMAO ternary mixture, respectively. The black line refers to the temperature of 273.15K, the red to 298.15K and the blue one to 323.15K.

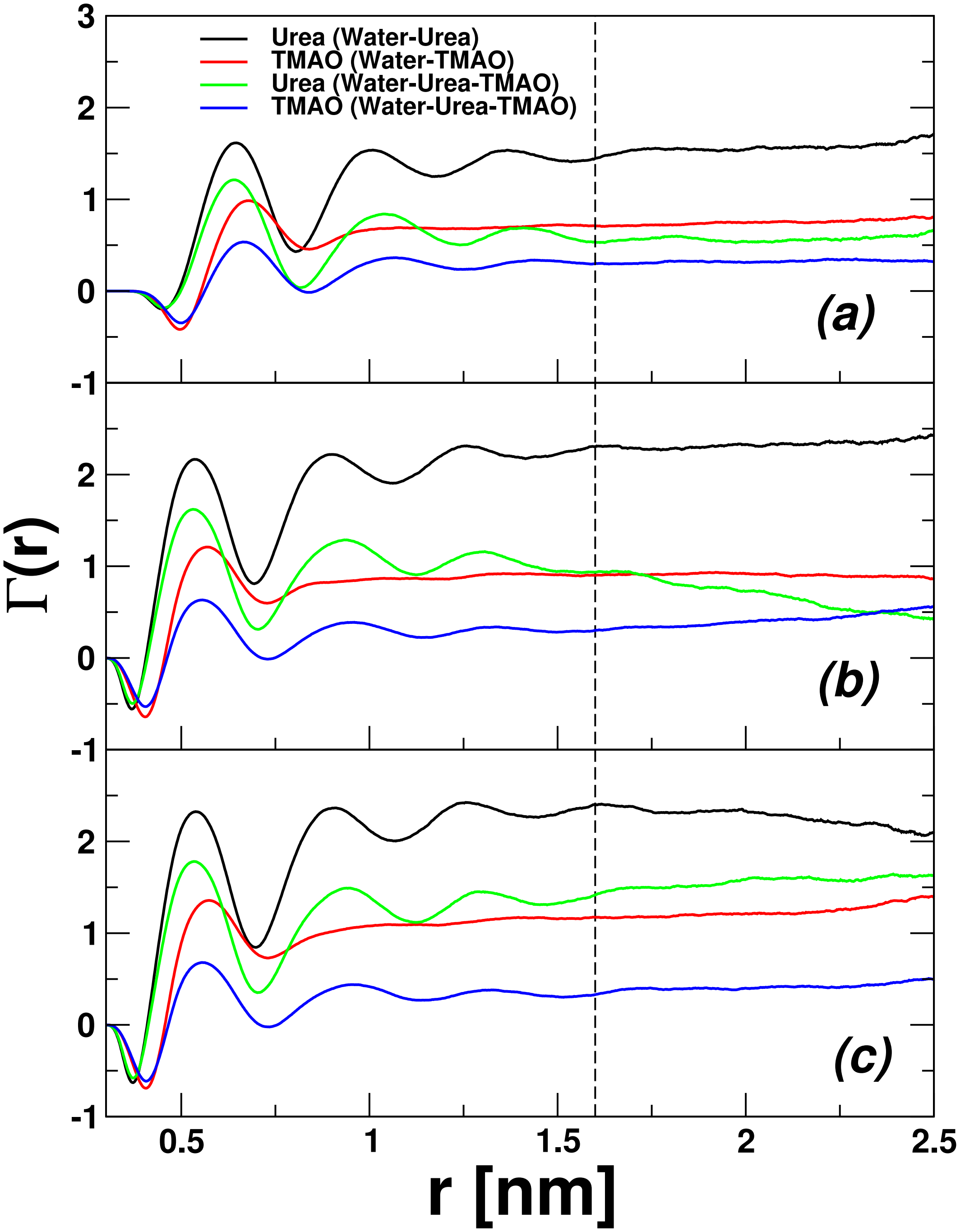

Figure SV: Preferential interaction (Γ) of urea and TMAO with each hydrophobic hydrocarbon investigated here at 298.15 K. Neopentane C_5H_{12} results are shown in (a), cyclohexane cC_6H_{12} one in (b) and of *n*-hexane nC_6H_{14} counterpart in (c). Black lines correspond to urea in 8.16M water-urea system, red lines to TMAO in 3.48M water-TMAO system, green and blue lines to urea and TMAO in ternary mixture water/7.18M urea/2.87M TMAO, respectively. The vertical intercepted line marks the border between the local and the bulk domains and correspond to the cutoff value used to determine Γ. A negative value of the total preferential binding coefficient Γ implies the depletion of the considered cosolute.

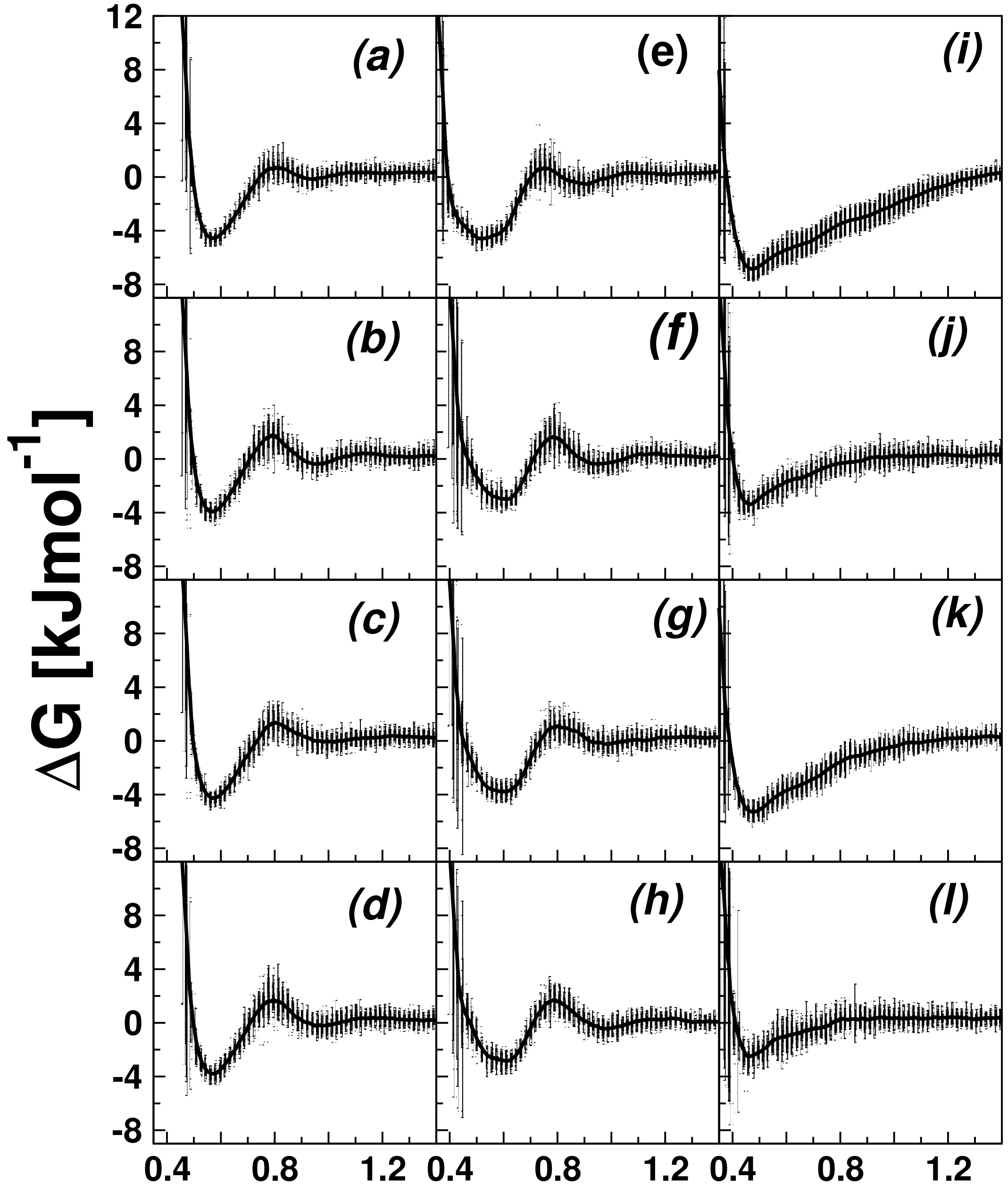

Figure SVI: Solute-solute Potential of Mean Force (ΔG) at 298.15 K for each of the hydrocarbons studied here in different solvent phases. Standard deviations are reported as vertical error bars. From left to right the results of neopentane C_5H_{12} (a)$\cdots$(d), cyclohexane cC_6H_{12} (e)$\cdots$(h) and *n*-hexane nC_6H_{14} (i)$\cdots$(l) are respectively displayed. From top to bottom results in different solvent mixtures namely pure water (a, e, i), 8.16M water-urea (b, f, j), 3.48M water-TMAO (c, g, k), and water/7.18M urea/2.87M TMAO (d, g, k) are respectively displayed.

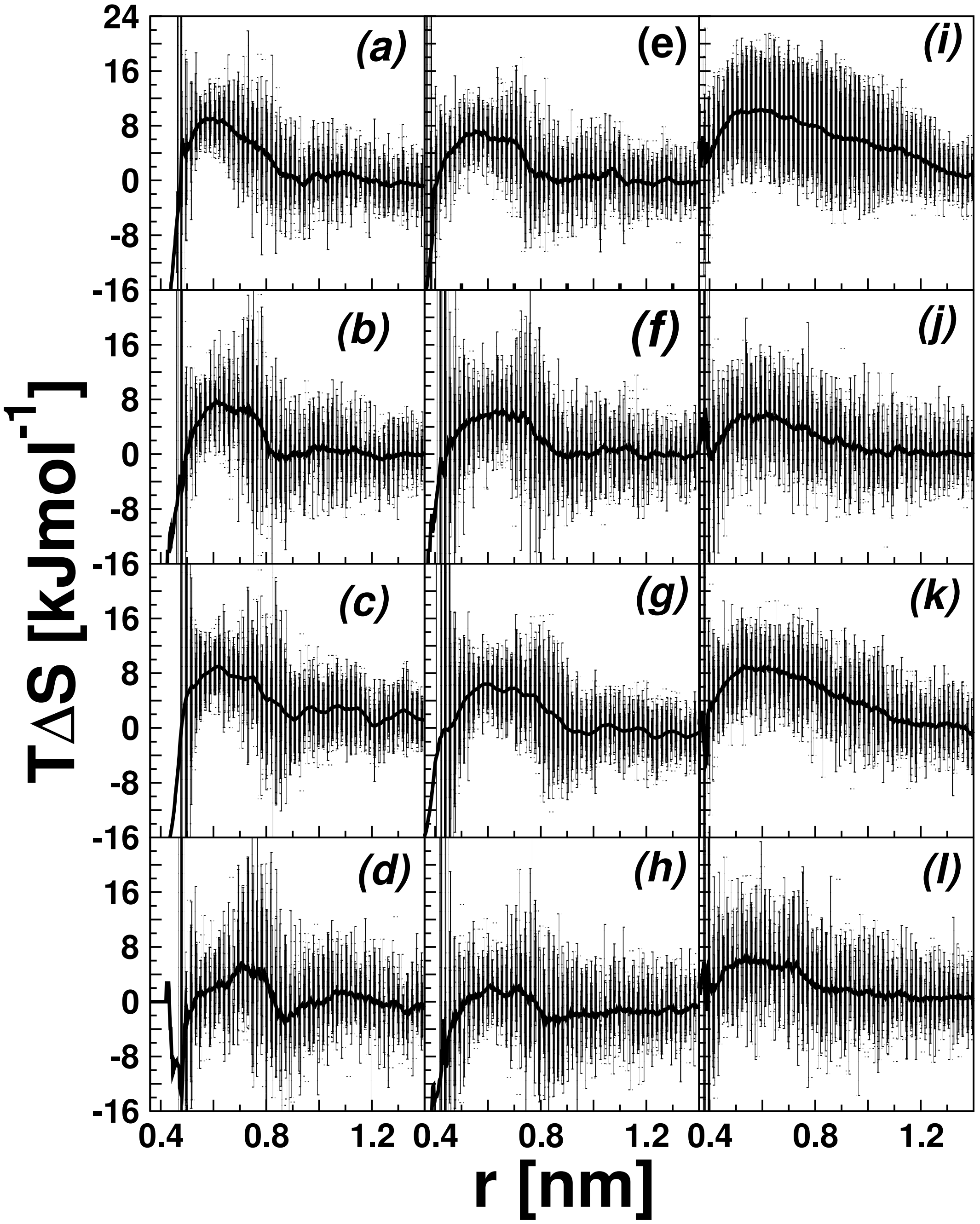

Figure SVII: Entropic contribution (TΔS) to the solute-solute Potential of Mean Force at 298.15 K for each of the hydrocarbons studied here in different solvent phases. Standard deviations are reported as vertical error bars. From left to right the results of neopentane C_5H_{12} (a)$\cdots$(d), cyclohexane cC_6H_{12} (e)$\cdots$(h) and *n*-hexane nC_6H_{14} (i)$\cdots$(l) are respectively displayed. From top to bottom results in different solvent mixtures namely pure water (a, e, i), 8.16M water-urea (b, f, j), 3.48M water-TMAO (c, g, k), and water/7.18M urea/2.87M TMAO (d, g, k) are respectively displayed.

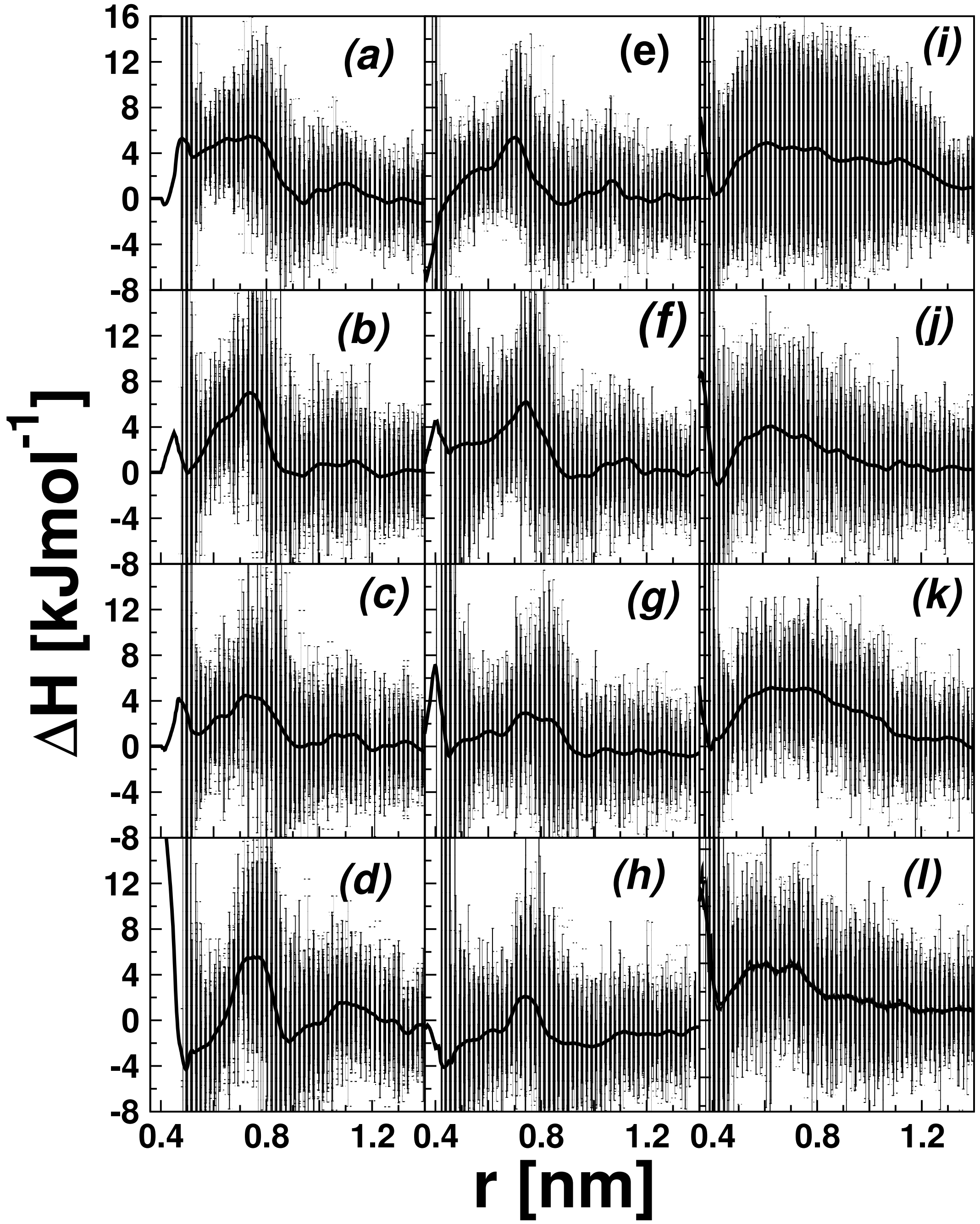

Figure SVIII: Enthalpic contribution (ΔH) to the solute-solute Potential of Mean Force at 298.15 K for each of the hydrocarbons studied here in different solvent phases. Standard deviations are reported as vertical error bars. From left to right the results of neopentane C_5H_{12} (a)$\cdots$(d), cyclohexane cC_6H_{12} (e)$\cdots$(h) and n-hexane nC_6H_{14} (i)$\cdots$(l) are respectively displayed. From top to bottom results in different solvent mixtures namely pure water (a, e, i), 8.16M water-urea (b, f, j), 3.48M water-TMAO (c, g, k), and water/7.18M urea/2.87M TMAO (d, g, k) are respectively displayed.

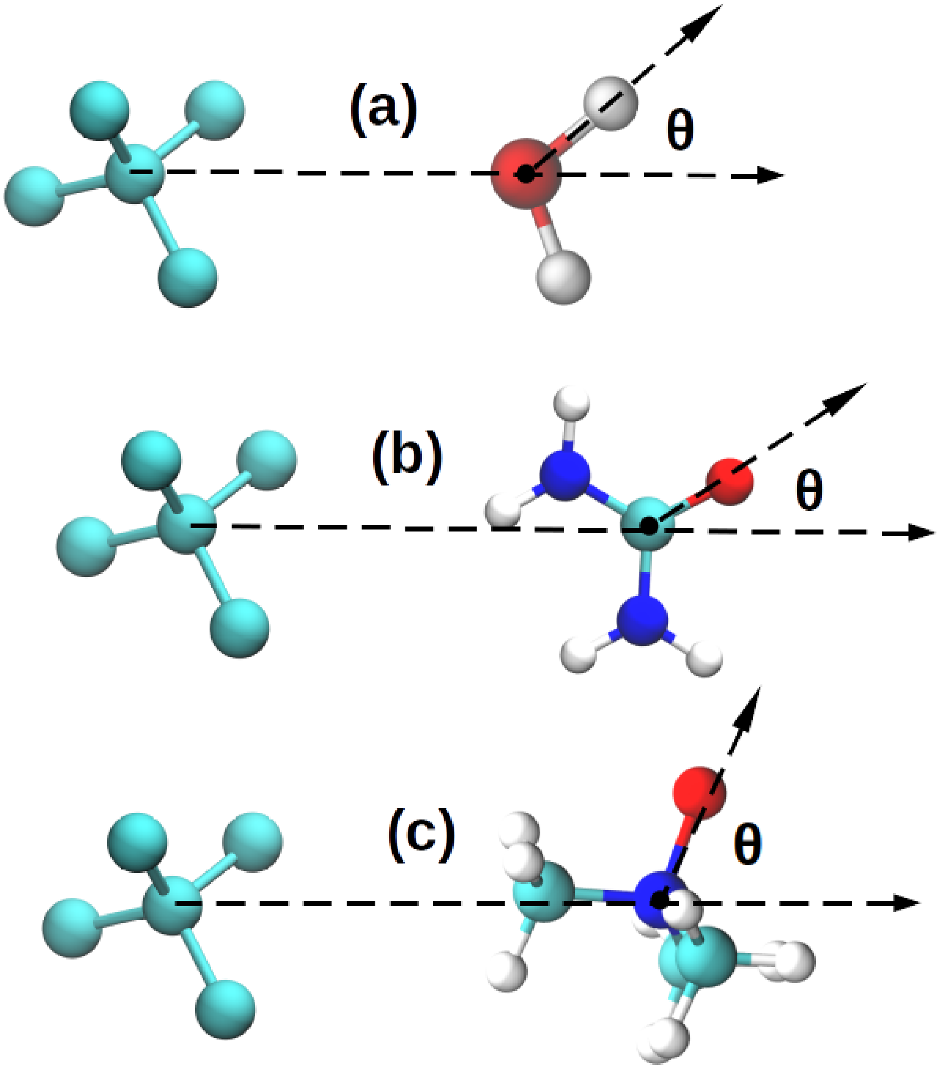

Figure SIX: Schematic illustration of the definition of the angle θ used to compute the angular distribution of co-solutes in the solvation shell in the case of neopentane. In (a) the angle formed between solute and water, in (b) between solute and urea and in (c) between solute and TMAO.

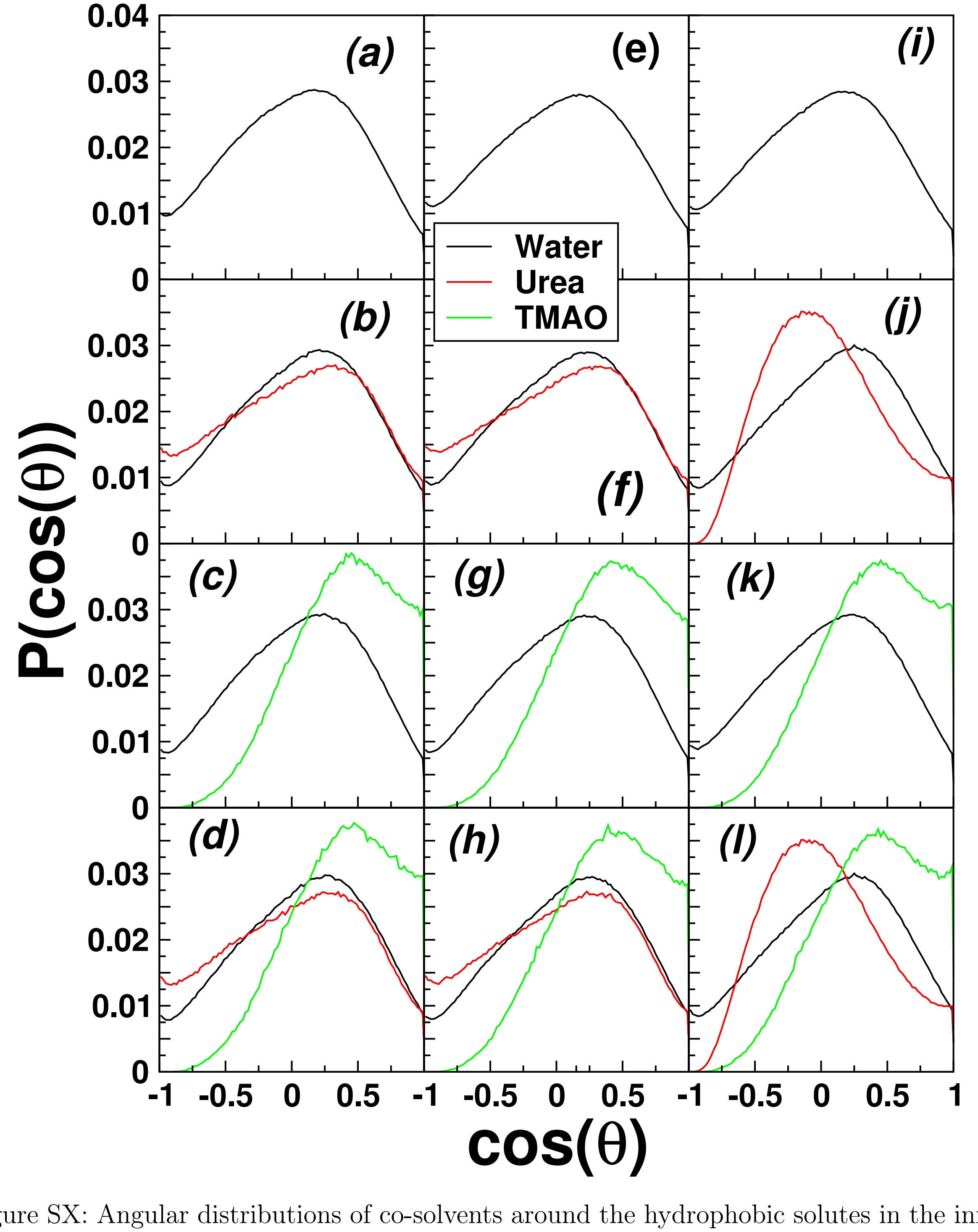

Figure SX: Angular distributions of co-solvents around the hydrophobic solutes in the inner solvation shell. The latter is taken here as the distance within 0.5 nm from the solute atoms. Co-solvents orientation around neopentane C_5H_{12} are shown on the left (a)$\cdots$(d), around cyclohexane cC_6H_{12} in the middle (e)$\cdots$(h), and around *n*-hexane nC_6H_{14} on the right (i)$\cdots$(l). From top to bottom panels show the results in water solution, 8.16M water-urea, 3.48M water-TMAO, and water/7.18M urea/2.87M TMAO ternary mixture, respectively. The black line refers to the angular distribution of water, red line to that of urea and green line to TMAO.

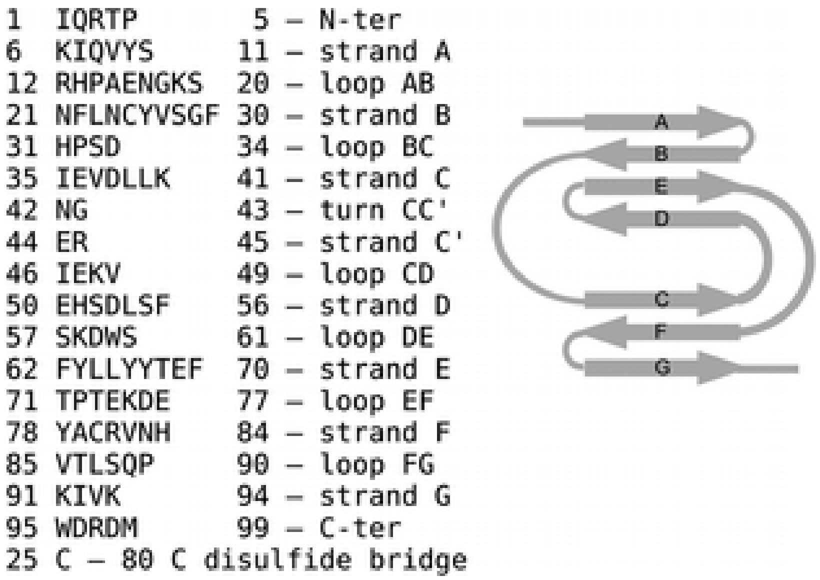

Figure SXI: Schematic view of the topology of β2m alongside with the secondary structure description.

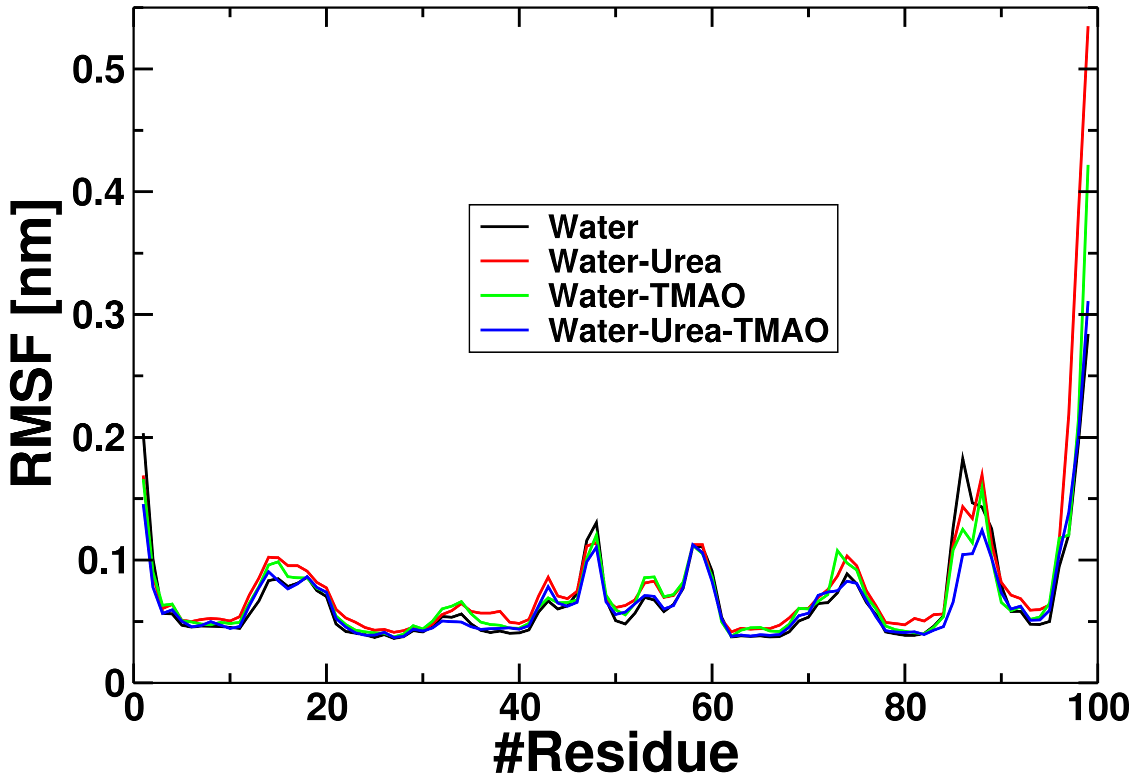

Figure SXII: Residue average root mean square fluctuations of backbone atoms. Black line depicts the simulation water, red line the one in 8.16M water-urea, while green line describes the simulation performed in 3.48M water-TMAO, and blue line shows the data obtained in ternary mixture water/7.18M urea/2.87M TMAO.

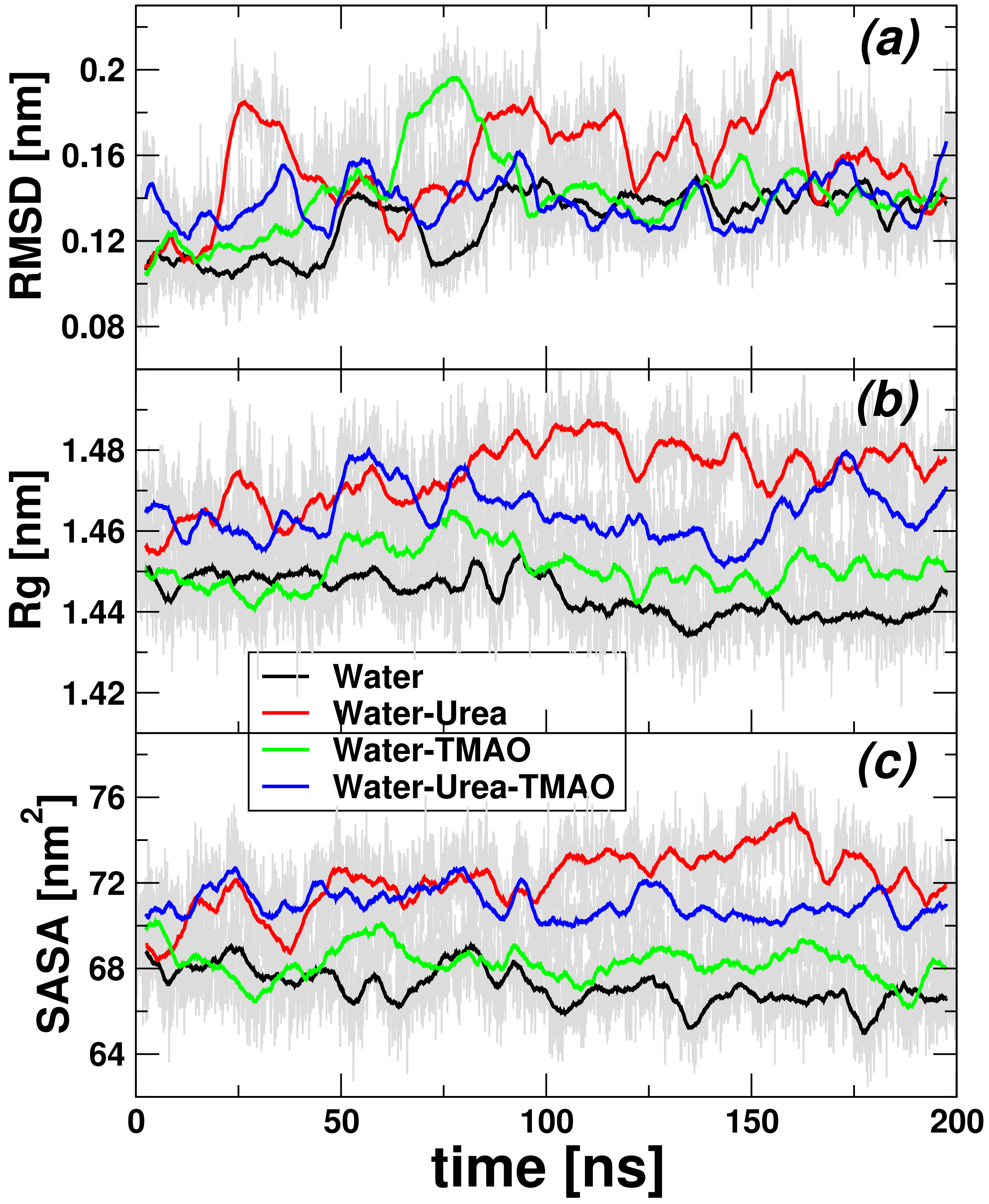

Figure SXIII: Conformational structural dynamics of β2m in different systems studied in this work. From top to bottom the time-dependent changes of the RMSD, Rg and SASA are displayed, respectively. Black line depicts the simulations in water, red line those in 8.16M water-urea, while green line describes the simulations performed in 3.48M water-TMAO, and blue line shows the data obtained in ternary mixture water/7.18M urea/2.87M TMAO.

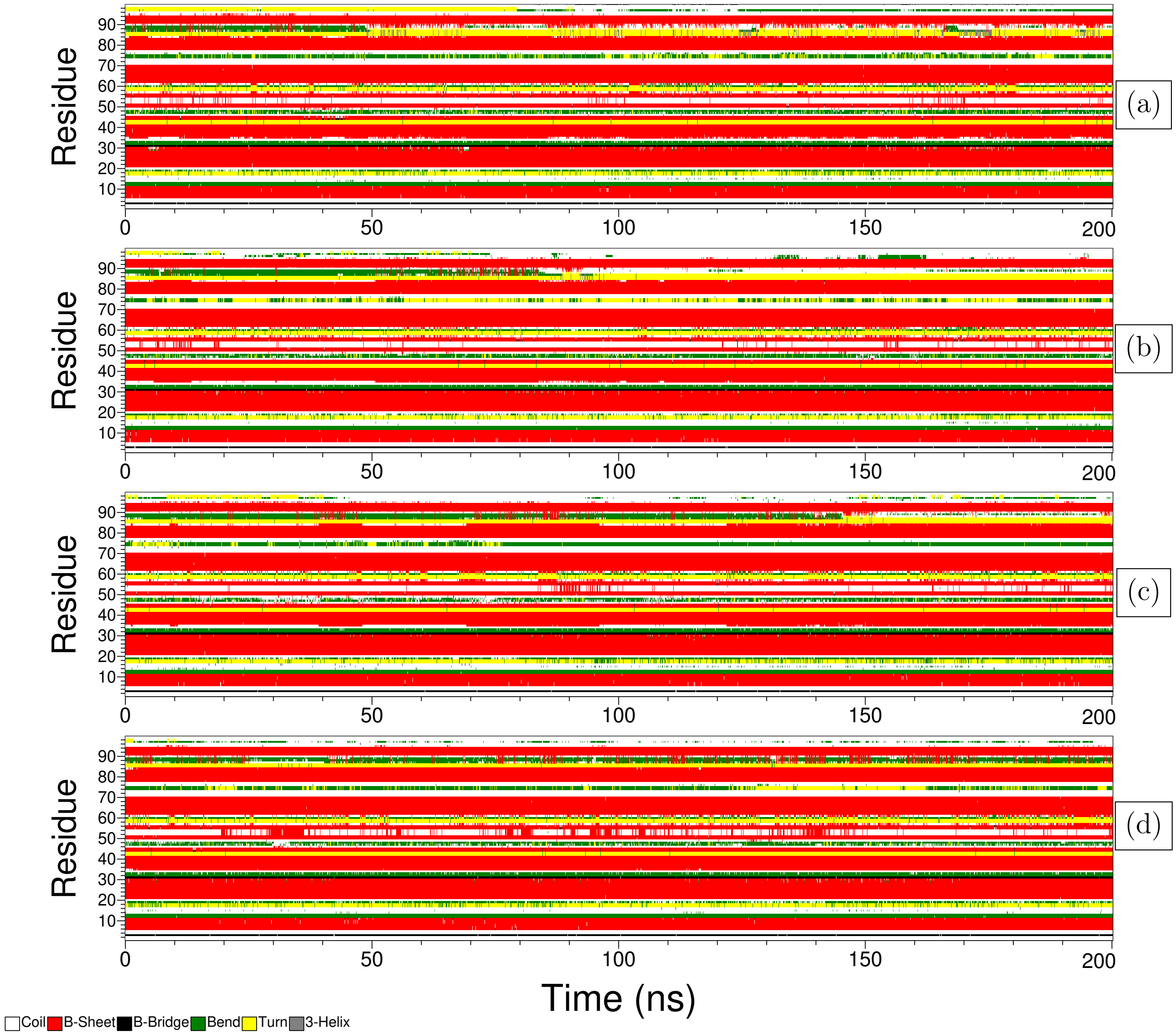

Figure SXIV: β2m secondary structure change as a function of simulation time. From top to bottom the secondary structure change of β2m in water H_2O (a), 8.16M water/urea (b), 3.48M water/TMAO (c) and ternary mixture water/7.18M urea/2.87M TMAO (d) solution are reported, respectively. Color coding for different secondary structural elements are shown at the bottom panel.
